# A simple urine test by 3D‐plus‐3D immunoassay guides precise *in vitro* cancer diagnosis

**DOI:** 10.1002/btm2.10489

**Published:** 2023-01-18

**Authors:** Hye Hyun Kim, Ok Jeong Moon, Yong Hwan Seol, Jeewon Lee

**Affiliations:** ^1^ Department of Chemical and Biological Engineering, College of Engineering Korea University Seoul Republic of Korea

**Keywords:** 3D‐IgG probes, 3D‐plus‐3D immunoassay, *in vitro* cancer diagnosis, signal self‐enhancement, urine test

## Abstract

Although a variety of urinary cancer markers are available for *in vitro* diagnosis, inherent problems of urine environment—containing various inorganic/organic ions/molecules that vary in concentration over a 20‐fold range or more and significantly attenuate antibody avidity for markers—render conventional immunoassays unsuitable, remaining unresolved and a major challenge. Here we developed a 3D‐plus‐3D (3p3) immunoassay method, based on a single‐step urinary marker detection by 3D‐antibody probes, which are free of steric hindrance and capable of omnidirectional capture of markers in a 3D solution. The 3p3 immunoassay showed an excellent performance in the diagnosis of prostate cancer (PCa) through detecting PCa‐specific urinary engrailed‐2 protein, demonstrating 100% sensitivity and 100% specificity with the urine specimens of PCa‐related and other related disease patients and healthy individuals. This innovative approach holds a great potential in opening up a novel clinical route for precise *in vitro* cancer diagnosis and also pushing urine immunoassay closer to more widespread adoption.

## 1 INTRODUCTION

Urine is a noninvasively, readily available body fluid that contains over 1500 proteins—approximately 70% comes from urinary organs and the urinary tract and the remaining 30% represents proteins filtered by glomerulus,^1^, ^2^ indicating that urine is a good source for *in vitro* diagnosis of various urogenital and systemic diseases including cancer. Among various body fluids such as serum, plasma, urine, and so on. urine is most favorable for point‐of‐care testing (POCT) that is a powerful platform for home testing, which enables individuals to monitor their health status through frequent diagnosis at their homes so that diseases can be diagnosed at the earliest possible time‐point.^3^, ^4^ In particular, it is worthy of noting that a variety of cancer markers (e.g., engrailed‐2 protein [EN2]^5^ or prostate‐specific antigen [PSA]^6^ for prostate cancer, bladder cancer‐specific nuclear matrix protein‐4 [BCLA‐4]^7^ for BCa, kidney injury molecule‐1 [KIM‐1]^8^ for renal cancer, human epididymis protein 4 [HE4]^9^ for ovarian cancer, trefoil factor [TFF1]^10^ for pancreatic cancer, a disintegrin and metalloprotease domain 12 [ADAM12]^11^ for gastric cancer, leucine‐rich‐alpha2‐glycoprotein 1 [LRG1]^12^, ^13^ for lung cancer, *etc*.) have been found in urine, and thus urine holds a great potential for POCT‐based, precise *in vitro* cancer diagnosis.^14^, ^15^, ^16^ However, unlike serum, urine contains a much more amount of various inorganic ions (K^+^, Na^+^, Cl^−^, Mg^2+^, and Ca^2+^) and organic molecules such as urea, uric acid, creatinine, hippuric acids, ascorbic acid, phenylacetylglutamine, and so on.^17^, ^18^, ^19^ and most of these components vary in concentration over a 20‐fold range or more depending on diet/fluid intake and other factors.^20^ This urine complexity and variability interfere with the interaction between disease‐specific urinary markers and their cognate antibodies (capture probes) for immunoassay and thereby render conventional immunoassays not suitable any more,^21^, ^22^, ^23^ requiring an advanced methodology that can resolve the urine‐associated problems.

A variety of immunoassay methods have been introduced for *in vitro* diagnosis, including molecularly imprinted polymer (MIP)‐based, electrochemiluminescence immunoassay‐based (ECLIA), and single‐molecule array (SIMOA)‐based detection methods.^24^, ^25^, ^26^, ^27^ However, the following main limitations remain unresolved yet. First, most immunoassays depend on two‐dimensional (2D) mode detection, that is, antibody probes—that are already immobilized on a 2D solid surface—capture disease markers in viscous serum, and thus the access of disease markers to the immobilized probes depends on only marker molecules' diffusional mobility. Furthermore, the immobilized probes are in general randomly oriented and thus sterically hindered on the 2D surface, often leading to poor diagnostic performance.^28^ For example, immunoglobulin G (IgG) probes need to be immobilized with such an orientation that Fc region is always perpendicular to the surface; however, which is not actually implementable. Although various chemical methods have been introduced to resolve the random orientation problem, they have been unsuccessful yet except for several complicated and costly methods.^29^ Finally, conventional immunoassays require tedious multiple steps for washing out nonspecifically, weakly bound false molecules and/or for adding prelabeled reporter probes, which significantly lowers diagnostic speed and accuracy and precludes convenient POCT. (Lateral flow assays are currently used in POCT but only for preliminary diagnostic assay due to low sensitivity and specificity.)

Due to these limitations, conventional 2D‐immunodetection cannot resolve the aforementioned urine‐associated problems as proven in this study. Here, we developed an innovative 3p3 immunoassay method, which is based on target marker detection by 3D‐IgG probes—which do not have a steric hindrance problem—in a 3D solution where both 3D‐IgG probes and disease markers move freely through omnidirectional diffusion, enhancing target accessibility to probes and thus leading to sensitive detection of urinary disease markers. The 3D‐IgG probe was constructed by attaching multiple IgG molecules on a single 3D scaffold—genetically modified capsid particle (~ 36 nm) of hepatitis B virus (HBV), consisting of 240 subunit proteins. That is, 240 copies of B domain of *Staphylococcal* protein A (SPA_B_) was genetically presented on the surface of a single HBV capsid particle, followed by attaching multiple IgG probes to the SPA_B_‐presenting capsid particle through specific and strong interaction between SPA_B_ and Fc region of IgG.^30^ Thereby, all antigen‐binding domains of IgG probes are subject to become fully available in capturing disease markers without steric hindrance. Furthermore, the 3p3 immunoassay is performed in a single step in an assay solution, requiring neither washing steps nor prelabeled reporter probes. Consequently, this 3p3 immunoassay is likely to have significant advantages in resolving the aforementioned problems of 2D immunoassays and thus overcoming the hurdles in urine immunoassay.

Here we applied the 3p3 immunoassay to the detection of urinary engrailed‐2 protein (EN2), a prostate cancer (PCa)‐specific marker. PCa has recently become a major health threat to men, surpassing the incidence rate of lung cancer but can be hardly prevented due to numerous risk factors such as aging, family history of prostate‐related diseases and *BRCA 1* or *2* gene mutations, and so on. The immunoassay of serum PSA that is excessively released from the prostate tissue damaged by carcinogenesis into the bloodstream through sloppy vascular tissues has significantly increased the detection rate of PCa over the past 30 years.^31^, ^32^, ^33^, ^34^, ^35^, ^36^ However, PSA is prostate tissue‐specific, not PCa‐specific, meaning that the PSA level also increases in case of other prostate diseases such as infectious and chronic inflammation, benign prostatic hyperplasia (BPH), and other tissue‐associated diseases near prostate.^37^, ^38^ Reportedly, only 20% of subjects with high PSA levels are finally diagnosed with PCa,^39^, ^40^, ^41^, ^42^ causing many unnecessary biopsies or treatment of individuals who do not have prostate malignancies,^43^, ^44^ and thus precise diagnosis of PCa is critical but yet remains unaccomplished. EN2 is a transcription factor involved in early embryo development of PCa and thus is only expressed in PCa cells and secreted into urine.^5^, ^45^, ^46^, ^47^, ^48^, ^49^, ^50^, ^51^, ^52^ The detection of urinary EN2 is currently based on ELISA, but the sensitivity and specificity are only 66%–80% and 80%–88%, respectively.^5^, ^45^, ^46^, ^47^ This study has demonstrated that the 3p3 immunoassay shows superior sensitivity and specificity in the urinary EN2 detection of PCa patients—no false‐negative signals for PCa patients (100% sensitivity) and no false‐positive signals for other prostate‐related diseases (BPH and BCa) and healthy controls (100% specificity)—indicating that this innovative approach is highly suitable as a novel immunoassay platform for simple urine test‐based, clinical *in vitro* cancer diagnosis.

## 2 RESULTS AND DISCUSSION

### 2.1 Prostate cancer marker: Prostate tissue‐specific marker (PSA) versus prostate cancer‐specific marker (EN2)

**FIGURE 1:**
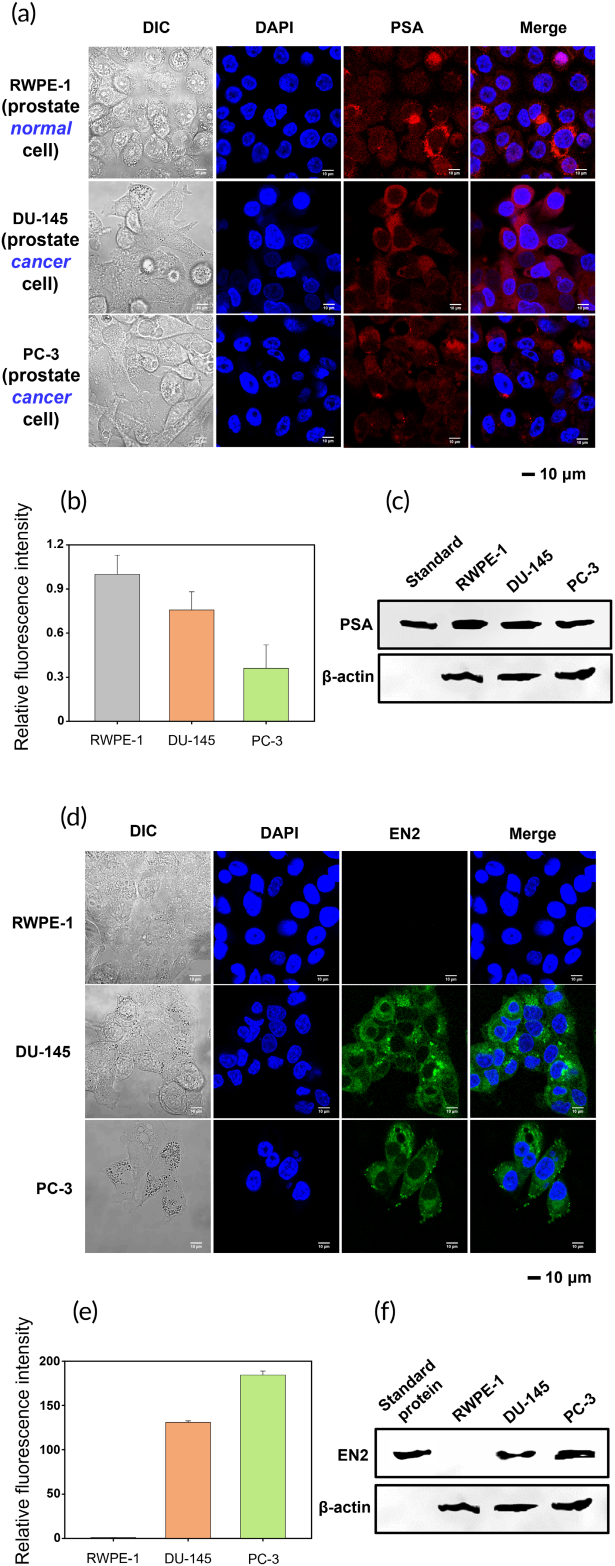
Expression of EN2 and PSA in prostate cancer‐ and normal cells. (a) Confocal microscopy images of prostate normal (RWPE‐1 [1 × 10^5^ cells/dish]) and cancer cells (DU‐145 [1 × 10^5^ cells/dish] and PC‐3 [1 × 10^5^ cells/dish]) treated with PE‐conjugated anti‐PSA antibodies (scale bar: 10 μm). (b) Relative fluorescence intensities of (a). (c) Results of immunoblotting of prostate normal cell (RWPE‐1) and cancer cell lysates (DU‐145 and PC‐3) using anti‐PSA antibodies. (d) Confocal microscopy images of prostate normal (RWPE‐1 [1 × 10^5^ cells/dish]) and cancer cells (DU‐145 [1 × 10^5^ cells/dish] and PC‐3 [1 × 10^5^ cells/dish]) treated with FITC‐conjugated anti‐EN2 antibodies (scale bar: 10 μm). (e) Relative fluorescence intensities of (d). (f) Results of immunoblotting of prostate normal cell (RWPE‐1) and cancer cell lysates (DU‐145 and PC‐3) using anti‐EN2 antibodies. In (b) and (d), relative fluorescence intensities were presented by regarding the intensity of RWPE‐1 as 1.0. In (c) and (f), β‐actin is a constitutively expressed protein in all types of cells (positive control). EN2, engrailed‐2 protein; FITC, fluorescein isothiocyanate; PE, phycoerythrin; PSA, prostate‐specific antigen

We investigated the expression of EN2 and PSA in normal prostate epithelium cells (RWPE‐1 [Cat. No. CRL‐11609], American Type Culture Collection [ATCC], Manassas, VA, USA) and prostate cancer cells (DU‐145, Cat. No. 30081, Korea Cell Line Bank [KCLB], Seoul, South Korea, and PC‐3, Cat. No. 21435, KCLB) through Western blotting. RWPE‐1, DU‐145, and PC‐3 cells were incubated with phycoerythrin (PE)‐labeled anti‐PSA antibody (Cat. No. 70‐XG69; Fitzgerald, Acton, MA, USA) and also with fluorescein isothiocyanate (FITC)‐labeled anti‐EN2 antibody (Cat. No. TA32493; Origene, Rockville, MD, USA), and the fluorescence from the cell cultures was analyzed using confocal fluorescence microscopy. It was found that PSA was expressed in both cancer and normal cell lines, and notably even a more amount of PSA was expressed in normal RWPE‐1 cells than in cancerous DU‐145 and PC‐3 cells (Figure 1a,b), indicating that PSA is not PCa‐specific at all. Western blot analysis of all cell lysates shows the same results as the confocal fluorescence microscopy (Figure 1c). These results exactly correspond to the previous reports that PSA is expressed in normal epithelial cells lining prostate wall and readily secreted into a blood vessels or urethra, depending on age and prostate size.^53^, ^54^, ^55^ The blood PSA level increases even under non‐malignant conditions such as prostatitis,^43^ and on the other hand, no PSA or a very little amount of PSA is often detected in the blood of PCa patients (Table S1). On the contrary to PSA, EN2 is expressed only in cancer cells (Figure 1d–f), indicating that EN2 is strictly PCa‐specific as previously reported,^5^, ^45^, ^46^, ^47^ and thus we confirm here that EN2 is a very promising biomarker for PCa diagnosis.

### 2.2 Construction of 3D‐IgG probes

The tandem repeat of SPA_B_ that has strong and specific affinity for Fc region of IgG (reportedly, K_d_ [dissociation constant] = 6.2 × 10^−10^ M) (named [SPA_B_]_2_) was genetically inserted into spike loop region of each subunit protein of HBV capsid (HBVC). Hexa‐histidine tag (H_6_) for both Ni^+2^‐affinity purification and Au^+3^ adsorption was also fused to the N‐terminus of each subunit protein. Considering that HBVC is comprised of 240 subunits, 240 copies of (SPA_B_)_2_ were subject to be presented on the outermost surface of HBVC, and the SPA_B_‐presenting HBVC—which was used as a 3D scaffold for constructing 3D‐IgG probes—was synthesized in recombinant *Escherichia coli* and subsequently purified through Ni^+2^‐affinity chromatography (Figure S1a,b). Transmission electron microscopy (TEM) and dynamic light scattering analyses of the purified SPA_B_‐presenting HBVC show native HBVC‐like particle formation with a narrow size distribution (Figure S1c,d). Next, anti‐human EN2 polyclonal IgG molecules were attached to the SPA_B_‐presenting HBVC particle via specific and strong interaction between SPA_B_ and Fc region of the IgG, resulting in the construction of anti‐EN2 3D‐IgG probe (Figure S1a), which was then stably stored after freeze‐dried. It is worthy of noting that all the anti‐EN2 IgG molecules of the 3D‐IgG probe has such an orientation that each Fc region is always perpendicular to the 3D scaffold surface, indicating that the 3D‐IgG probes have no steric hindrance problem in capturing EN2.

### 2.3 Performance of 3p3 immunoassay in urinary EN2 detection

#### 2.3.1 Limitation of conventional immunoassays in urinary protein detection

**FIGURE 2:**
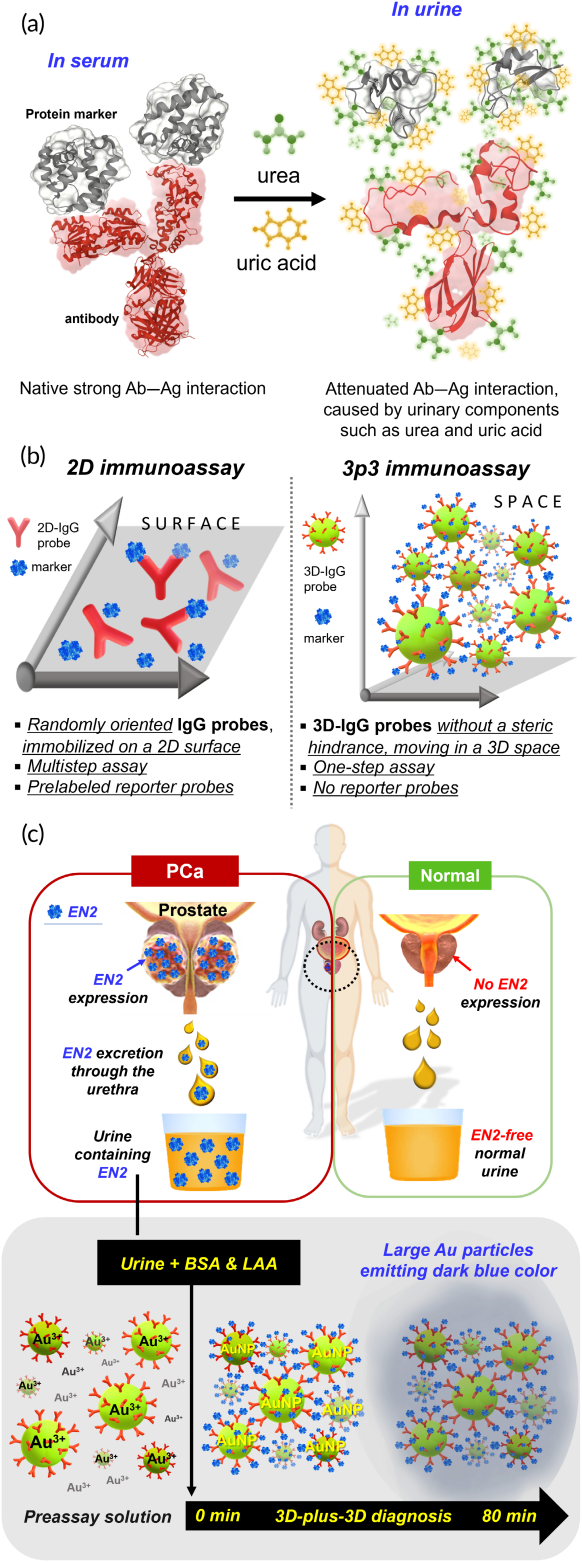
Schematic illustration of 3p3 immunoassay. (a) Interaction between antibody probe (red) and protein disease markers (gray) in serum and urine, showing denatured conformation of antibodies and protein markers and altered hydrogen and ionic bonds by urea (green) and uric acid (yellow) in urine. (b) Comparison of important characteristics between 2D and 3p3 immunoassay. (c) 3p3 immunoassay‐based PCa diagnosis through detecting PCa‐specific urinary EN2. 3p3, 3D‐plus‐3D; PCa, prostate cancer

The aforementioned urine complexity and variability interfere with the interaction between disease‐specific urinary markers and their cognate IgG probes. In particular, a high concentration of urea and uric acid in urine significantly attenuated the avidity of IgG probes for urinary marker proteins (Figure 2a). Uric acid that lowers pH of urine affects both hydrogen bonds between and charge properties of amino acid residues and thereby weakens the interaction between protein markers and IgG probes. For example, at low urine pH (often below 6.0), histidine (pK_R_ ≈ 6.0) charge is altered, and overall net charge of glutamate residues (pK_R_ ≈ 4.3) could be less negative in urine than in serum. In addition, a large amount of urea not only denatures the conformation of IgG and protein markers but also interrupts hydrogen bonds. Therefore, this unique urine environment renders conventional 2D immunoassays (e.g. ELISA, ECLIA, *etc*.) not any more suitable (Figure 2b).

#### 2.3.2 Principle and applicability of 3p3 immunoassay

3p3 immunoassay is based on the use of 3D‐IgG probes in a 3D space (an assay solution) (Figure 2b). Steric hindrance‐free 3D‐IgG probes and disease markers move freely through omnidirectional diffusion, which remarkably enhances marker accessibility to probes and thus sensitivity. As illustrated in Figure 2c, the 3p3 immunoassay for detecting urinary EN2 is initiated just by adding PCa‐patient urine, l‐ascorbic acid (LAA, reducing agent) and bovine serum albumin (BSA) to a premade assay solution (i.e. a preassay solution containing anti‐EN2 3D‐IgG probes adsorbed by gold ions [Au^3+^]), which is quickly followed by three simultaneous events: (1) EN2‐dependent clustering of the 3D‐IgG probes, (2) reduction of adsorbed Au^3+^, and (3) formation of large gold particles—actually large clusters of gold nanoparticles (AuNPs)—emitting dark blue color. Because only true marker molecules (EN2) can make such a quick formation of large clusters of 3D‐IgG probes, there is no need of washing steps to remove false molecules, indicating a single‐step, sensitive in vitro diagnosis of prostate cancer.

**FIGURE 3:**
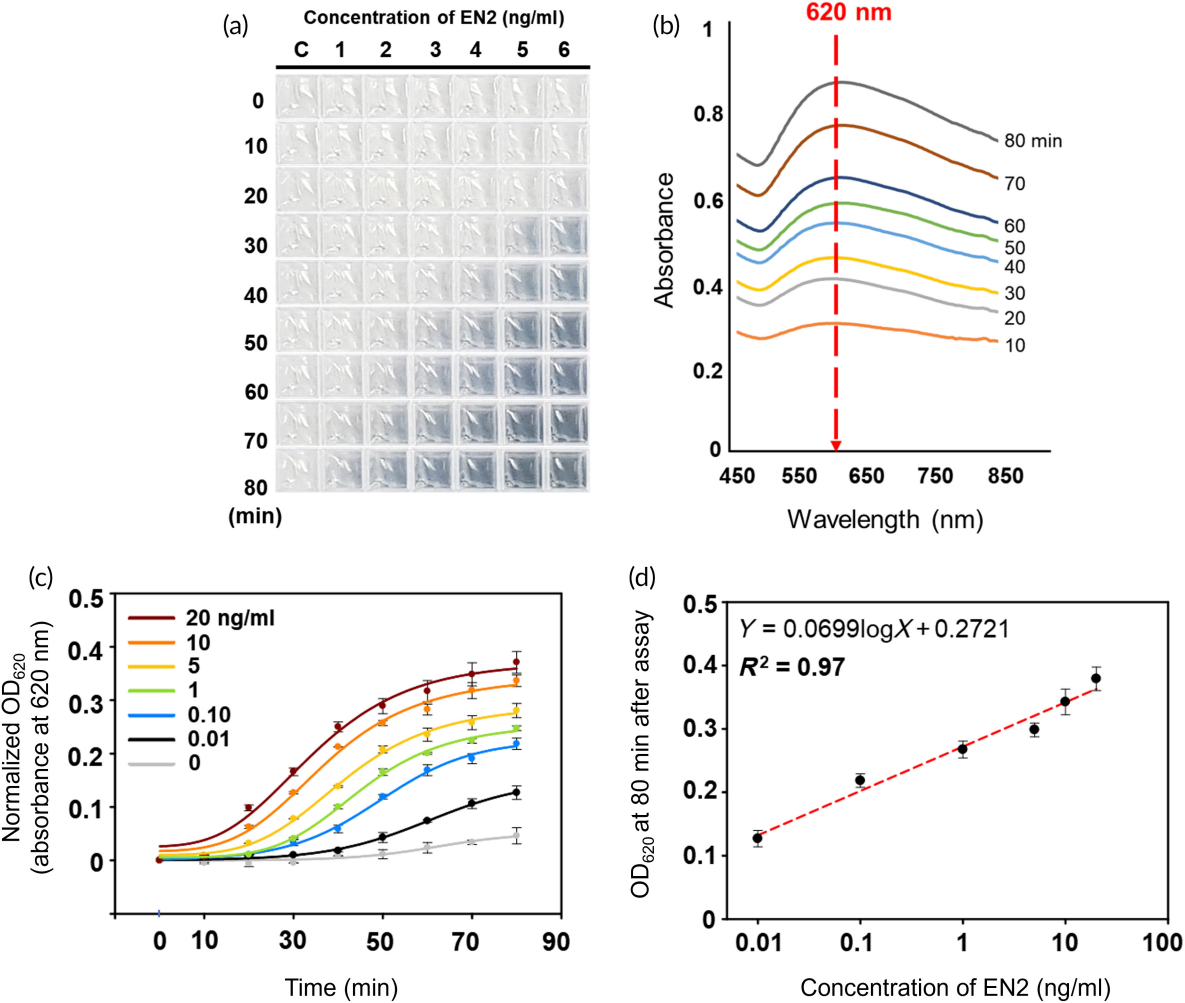
3p3 immunoassay of standard EN2 spiked in healthy urine. (a) Photographic images of 384‐well plates, showing time‐course change of optical assay signals in the 3p3 immunoassay performed with urine samples of standard EN2 at different concentrations (C: 0 ng/ml, 1: 0.01 ng/ml, 2: 0.1 ng/ml, 3: 1.0 ng/ml, 4: 5.0 ng/ml, 5: 10 ng/ml, 6: 20 ng/ml). (b) Time‐course change of light absorption spectra of Sample 6 in (a), showing that maximum light absorption happens always at 620 nm. (c) Time‐course change of OD_620_ measured for the optical assay signals of (a). (d) OD_620_ measured at 80 min after the EN2 detection by 3p3 immunoassay of (a) begins. 3p3, 3D‐plus‐3D; EN2, engrailed‐2 protein

First, the 3p3 immunoassay was performed with standard urine samples containing commercial EN2 standard (i.e., healthy female urines spiked with recombinant human EN2; Cat. No. TP‐311220; Origene) at the predetermined concentrations (0.01–20 ng/ml). Each standard urine sample (4 μl) was mixed with BSA (13%w/v, 4 μl) and LAA (0.7%w/v, 8 μl), and the resulting mixture was added to a preassay solution (24 μl) in each well of 384‐well plate, which contains anti‐EN2 3D‐IgG probes (10 mg/L) where Au^3+^ was already adsorbed through coordination bond with H_6_ (Figure S1a, Section 4). As shown in Figure 3a, a dark blue color appeared more rapidly as the EN2 concentration increases from 0.01 to 20 ng/ml. Also, it is notable that for the EN2‐free healthy control, any discernable color change was not observed even at 80 min after the 3p3 immunoassay began. The analyses of TEM and energy‐dispersive X‐ray (EDX) spectroscopy confirm that the dark blue color comes from large Au particles, that is, the larger the size of Au particles is, the darker the blue color becomes (Figure S1e,f). Interestingly, light absorption spectra shows that as the color gradually darkens the maximum absorbance always occurs at a wavelength of 620 nm (*λ*_max_) (Figure 3a,b), which is in good agreement with the “raspberry” model^56^, ^57^ that was established to explain optical properties of gold particles (i.e., clusters of AuNPs). According to this model, larger clusters of AuNPs possess a larger absorption cross section per cluster, while resonance wavelength remained nearly constant. That is, exact morphological configuration is not critically important to the profile of absorption spectra of gold particles. Therefore, in the 3p3 immunoassay, it is likely that raspberry‐like large clusters of AuNPs are formed on the surface of 3D‐IgG probe‐marker complexes and absorb the visible light maximally at an identical wavelength irrespective of their size. The time‐course change in absorbance at 620 nm (OD_620_) for the standard urine samples shows that the OD_620_ signals are highly dependent on EN2 concentration for the entire period of assay (80 min) (Figure 3c), and the most linear correlation (*R* ^2^ = 0.97) between OD_620_ and EN2 concentration was achieved at 80 min (Figure 3d). From Figure 3c,d, the limit of detection (LOD) looks likely 10 pg EN2/ml urine, low enough to detect urinary EN2 of PCa patients.

The same 3p3 immunoassay was also performed with standard PSA samples of urine (i.e., healthy female urines spiked with commercial standard of human PSA, Cat. No. 30C‐CP1017U, Fitzgerald) using anti‐PSA 3D‐IgG probe. Figure S2a–d shows the equally excellent assay performance. The maximum absorbance occurred at a wavelength of 585 nm, and the most linear correlation (*R* ^2^ = 0.97) between OD_585_ and PSA concentration was also achieved at the same time point, 80 min after the assay began. It seems that the difference in *λ*_max_ in the EN2 and PSA detection arises in part from the difference in the content of lysine, which exerts a strong influence on *λ*_max_ of AuNPs. Reportedly, when attached on protein surface, AuNPs are stabilized by the electrostatic interaction between negatively charged AuNP surface and protonated ε‐amine group of lysine residues, leading to the red‐shift of absorption spectra of AuNPs.^58^, ^59^, ^60^ Thus, upon the close interaction of EN2 or PSA with AuNPs on the 3D‐IgG probe‐marker complexes, the lysine‐rich EN2—having 22 lysine residues as DNA‐binding motif^61^—is subject to causing more red‐shift of *λ*_max_ compared to PSA with 11 lysine residues.

#### 2.3.3 Superiority of 3p3 immunoassay over 2D immunoassay in urinary EN2 detection

**FIGURE 4:**
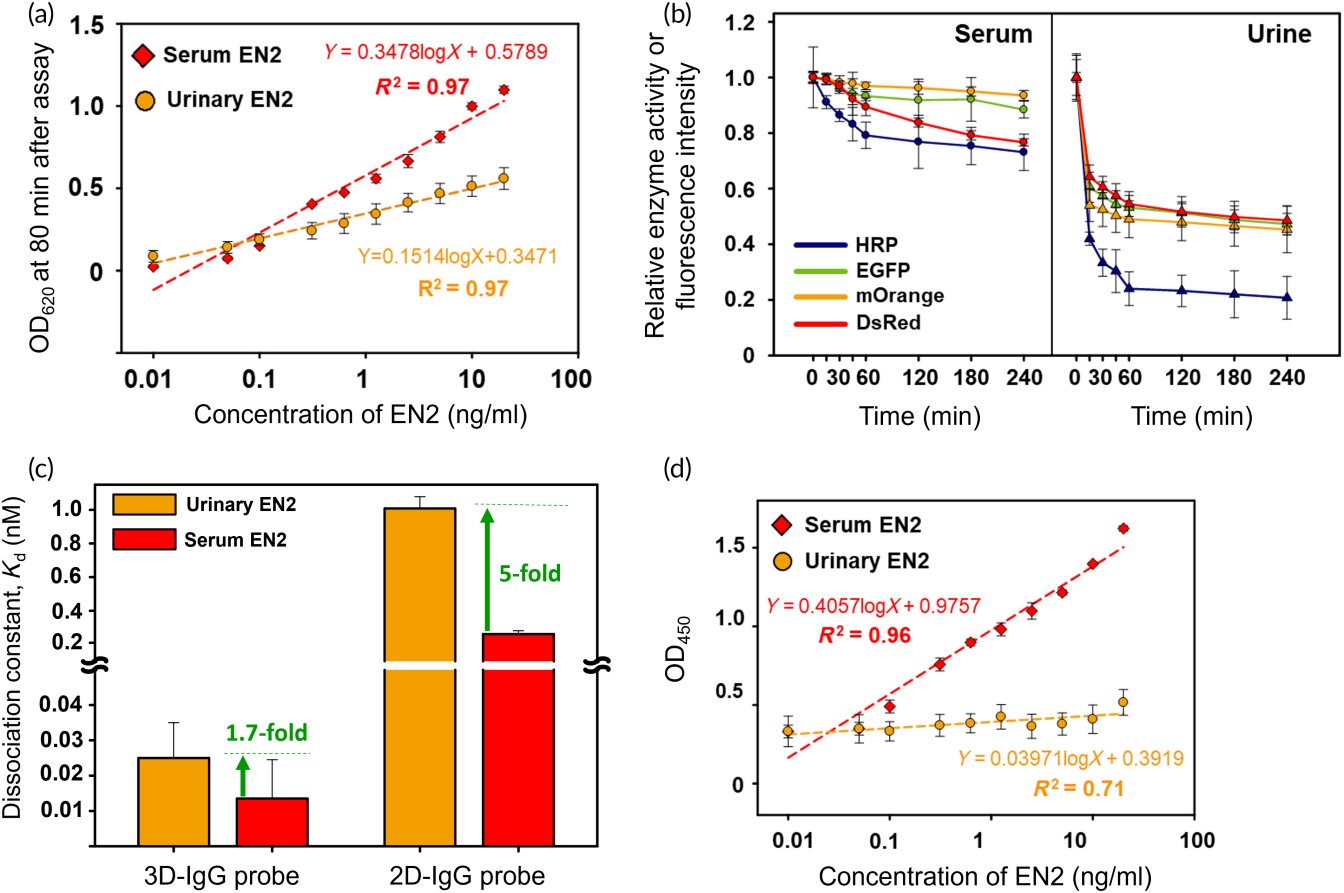
3p3 and 2D immunoassay performance in urine and serum. (a) Results of 3p3 immunoassay of standard EN2 in urine and serum. (b) Time‐course change of enzyme (HRP) and fluorescent protein (EGFP, mOrange, and DsRed) activities in urine and serum, which was estimated by measuring OD_450_ of enzymatic reaction solution and fluorescence intensity, respectively. Fluorescence intensity was measured at the following excitation (Ex) and emission (Em) wavelengths: EGFP (Ex/Em: 475/509 nm), mOrange (Ex/Em: 520/562 nm), and DsRed (Ex/Em: 541/596 nm). Relative activity/intensity was calculated by considering the activity/intensity value at 0 min as 1.0. (c) Dissociation constants (*K*_d_) estimated in the binding between EN2 and anti‐EN2 IgG probes (3D‐IgG and 2D‐IgG probe) under serum and urine environment. 2D‐IgG probe means the IgG probe immobilized on a 2D surface. (d) Results of ELISA of standard EN2 in urine and serum. 3p3, 3D‐plus‐3D; EN2, engrailed‐2 protein; HRP, horse radish peroxidase; IgG, immunoglobulin G; PSA, prostate‐specific antigen

The performance of 3p3 and 2D immunoassays was compared in the detection of EN2 (0.01–20 ng/ml) in serum and urine. The EN2 detection signals (OD_620_) of 3p3 immunoassay were measured at postassay 80 min as described above, and a commercial ELISA kit for EN2 (ELISA‐EN2) (Cat. No. OKCD09228; Aviva Systems Biology, San Diego, CA, USA) was used as a 2D immunoassay as per the supplier's instructions. Notably from Figure 4a, the 3p3 immunoassay detected both urinary and serum EN2 with excellent signal linearity. As the concentration exceeds 0.1 ng/ml, urinary EN2 gives lower absorbance signals than serum EN2 at each concentration, which is most likely because under urine environment protein conformation/function is significantly damaged, as demonstrated in Figure 4b, showing that the proteins (horse radish peroxidase [HRP] and green, orange, and red fluorescent proteins [EGFP, mOrange, and DsRed, respectively] with a stable β‐barrel structure) rapidly lose their own function in urine. The absorbance signals of urinary EN2 were augmented simply by using a more amount of 3D‐IgG probe (Figure S3), demonstrating another important advantage of 3p3 immunoassay that the quantity of 3D‐IgG probe is readily controllable unlike 2D immunoassays, because it is quite difficult to control the amount of immobilized IgG probes on a 2D surface.

The *K*_d_ values of 3D‐IgG and 2D‐IgG probes—defined as IgG probe used in 2D immunoassays—were estimated in the binding of EN2 to IgG probes in urine and serum, after the both probes are immobilized on each well of 96‐well plate (high‐binding plate, Cat. No. 44‐2404‐21; Thermo Fisher Scientific, Waltham, MA, USA) (Section 4). Figure 4c shows that 3D‐IgG probe has much higher avidity for both urinary and serum EN2 than 2D‐IgG probe. In particular, it is worthy of noting that the EN2‐binding avidity of both probes reduces when serum is switched with urine, as seen in the following *K*_d_ values: 0.013 (serum EN2) and 0.025 nM (urinary EN2) for 3D‐IgG probe and 0.260 (serum EN2) and 1.010 nM (urinary EN2) for 2D‐IgG probe, indicating that the avidity reduction fold is much smaller in 3D‐IgG probe (1.7‐fold) than in 2D‐IgG probe (5‐fold). This is because of the aforementioned advantage of 3D‐IgG probe (Figure 2b) over 2D‐IgG probes—all IgG molecules in 3D‐IgG probe bind to antigens without a steric hindrance, thus keeping high antigen‐capturing capacity even under urine environment. Of course, in this context, 2D immunoassay (ELISA‐EN2) shows a significant limitation in the urinary EN2 detection (Figure 4d).

### 2.4 Performance of 3p3 immunoassay in PCa diagnosis using patient urine specimens

#### 2.4.1 PCa diagnosis: “3p3‐ *vs*. 2D immunoassay” and “urinary EN2‐ *vs*. PSA detection”

**FIGURE 5:**
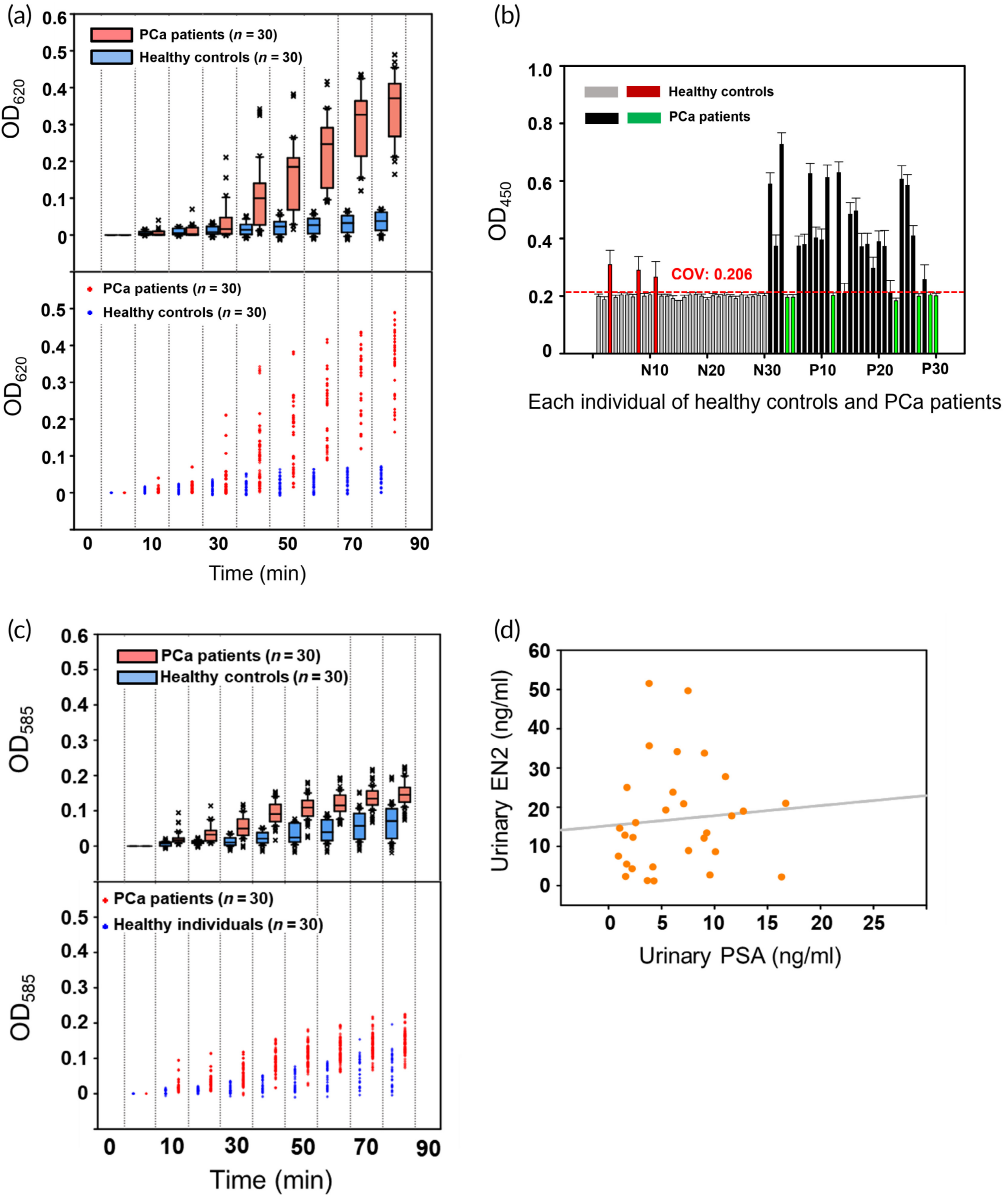
PCa diagnosis through detecting EN2 and PSA in PCa patient urine. (a) Results of 3p3 immunoassay‐based PCa diagnosis through EN2 detection using the urine of 30 PCa patients and 30 healthy individuals, presented in box and dot plots. (b) Results of ELISA‐based PCa diagnosis through EN2 detection using the urine of 30 PCa patients and 30 healthy individuals. COV represent cut‐off‐value, calculated as per instructions from ELISA kit supplier. False‐positive and ‐negative signals are marked by red and green bars, respectively. (c) Results of 3p3 immunoassay‐based PCa diagnosis through PSA detection using the urine of 30 PCa patients and 30 healthy individuals, presented in box and dot plots. (d) Correlation between urinary EN2 and PSA levels, indicating a very low Spearman's rank correlation coefficient (*ρ* = 0.176). 3p3, 3D‐plus‐3D; EN2, engrailed‐2 protein; PCa, prostate cancer; PSA, prostate‐specific antigen

As per the aforementioned procedure of 3p3 immunoassay using anti‐EN2 3D‐IgG probe, PCa diagnosis was performed with 30 patients' and 30 healthy urine specimens (negative controls) (Table 1), and only a small volume of urine (4 μl) was used in a 384‐well plate. Optical signals appeared clearly from all the urine samples of 30 PCa patients, while any discernible signals were not observed for 30 healthy controls (Figure S4a,b). Time‐course absorbance (OD_620_) change demonstrates that the assay signals between all PCa patients and healthy controls were apparently differentiated at 80 min after the diagnosis began (Figure 5a). (Using a predetermined linear correlation between OD_620_ and EN2 concentration [Figure 3d], OD_620_ at the 80 min was quantitatively converted to EN2 concentration for all the urine samples [Table S2a,b].). Surprisingly, this simple urine test shows superior sensitivity and specificity—generation of no false‐negative signals from all PCa patients (100% sensitivity) and no false‐positive signals from all healthy controls (100% specificity). On the other hand, the PCa diagnosis by ELISA‐EN2 using the same patient and healthy urine specimens shows seven false‐negative signals (76.7% sensitivity) and three false‐positive signals (90.0% specificity) (Figure 5b and Table S2c,d).

**TABLE 1:** Demographics of urine donors and summary of EN2 and PSA concentration in the donated urine samples, estimated through 3p3 immunoassay.

Group	*n*	Mean age (range)	Mean urinary PSA (ng/ml) (range)	Mean urinary EN2 (ng/ml) (range)
Healthy controls	30	70 (56–89)	0.926 (0.000–0.014)	0
Prostate cancer	30	75 (53–87)	6.383 (0.960–16.745)	16.911 (1.121–51.450)
BPH	15	68 (45–80)	13.165 (0.756–21.973)	0
Bladder cancer	15	72 (59–88)	7.331 (0.391–21.646)	0

Abbreviations: 3p3, 3D‐plus‐3D; BPH, benign prostatic hyperplasia; EN2, engrailed‐2 protein; PCa, prostate cancer; PSA, prostate‐specific antigen.

Furthermore, with the same patient and healthy urine specimens, PCa diagnosis was performed through the 3p3 immunoassay using anti‐PSA 3D‐IgG probe (Figure 5c, Table S3a,b). Time‐course absorbance (OD_585_) signals were never clearly differentiated between PCa patients and healthy controls during the entire period of assay (80 min), which is because healthy urine also has a detectable amount of PSA (Table S3b). The concentrations of EN2 and PSA in the urine samples of 30 PCa patients (Tables S2a and S3a)—which were all estimated through the 3p3 immunoassay—are plotted in Figure 5d, showing a very weak correlation (i.e., a Spearman's rank correlation coefficient of 0.176) between urinary EN2 and PSA and thus indicating again that PSA is not a reliable PCa marker. The PCa diagnosis was also performed using ELISA‐PSA (Cat. No. MBS355412; MyBiosource, San Diego, CA, USA) with the same patient and healthy urine specimens, resulting in nine false‐negative signals and two false‐positive signals (Figure S4c and Table S3c,d). Consequently, all the results above indicate that the 3p3 immunoassay can detect urinary EN2 with superior sensitivity and specificity and thereby enables precise PCa diagnosis. Furthermore, as shown in Figure S5, another important reason for the superiority of 3p3 immunoassay over ELISA in the urinary marker detection is that the use of much less amount of urine sample (4 μl) makes the 3p3 immunoassay less influenced by urea and uric acid and thus more sensitively detect the urinary marker compared to conventional 2D immunoassay like ELISA that uses in general much larger amount of sample (mostly 100 μl).

#### 2.4.2 Diagnostic selectivity: PCa *vs*. other related diseases

**FIGURE 6:**
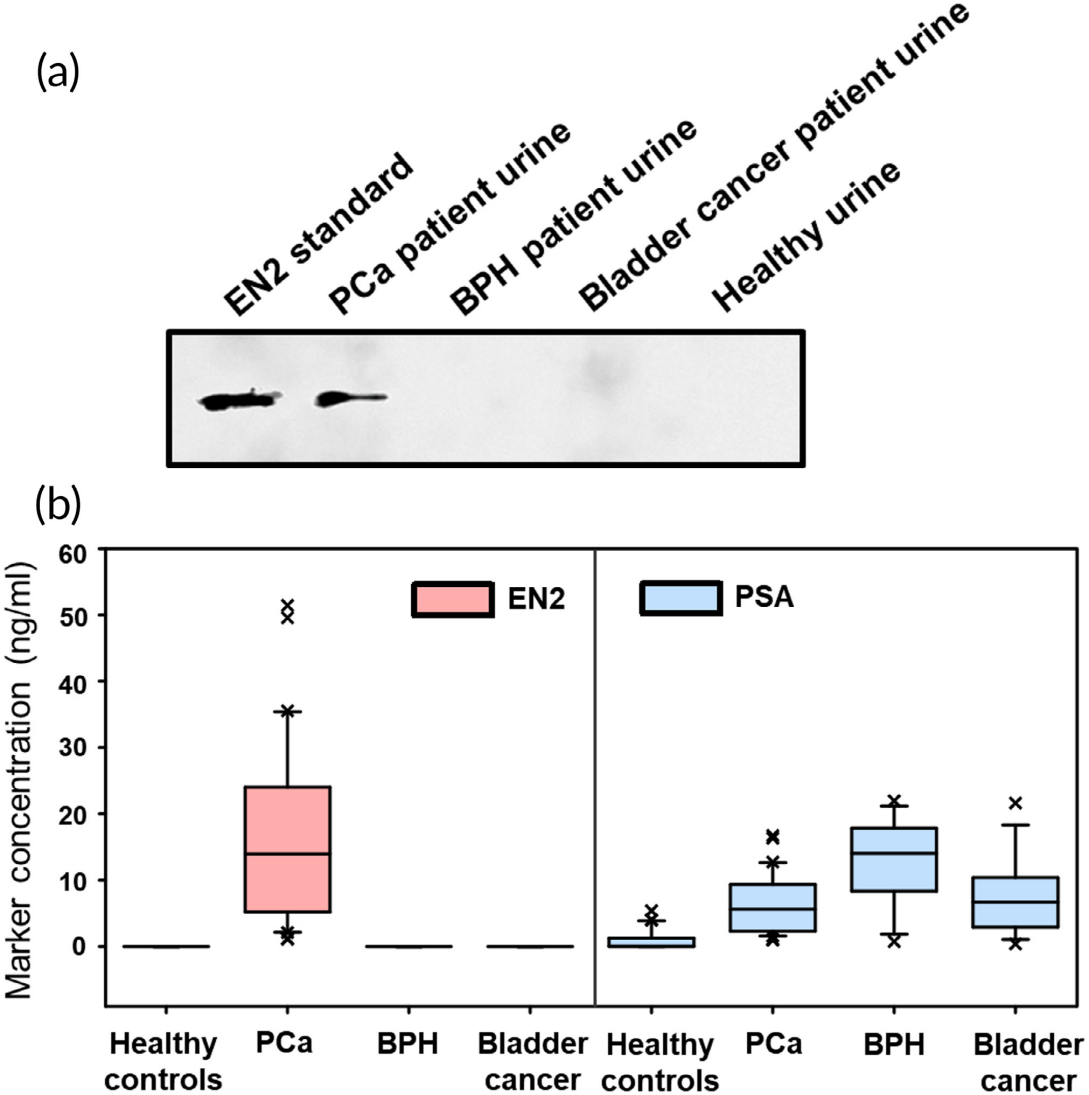
Diagnostic selectivity of 3p3 immunoassay in urinary EN2 detection. (a) Result of immunoblotting analysis to detect EN2 in the urine of PCa, BPH, and BCa patient and healthy individual. (b) Comparison of diagnostic results by 3p3 immunoassays using the urine of 30 PCa, 15 BPH, and 15 BCa patients and 30 healthy individuals: EN2 versus PSA detection, presented in box plot, showing excellent selectivity of PCa diagnosis performed through urinary EN2 detection‐based 3p3 immunoassay. 3p3, 3D‐plus‐3D; BPH, benign prostatic hyperplasia; EN2, engrailed‐2 protein; PCa, prostate cancer; PSA, prostate‐specific antigen

A precise diagnosis requires clear discrimination not only between disease and normal conditions but also between disease and related other diseases with similar symptoms. Reportedly, PCa diagnosis based on the detection of serum PSA frequently gives PCa‐positive signals even for other related disorders such as BPH and BCa that have common PCa‐like symptoms. Western blot analysis shows that EN2 is found only in the urine of PCa patient, not in BPH and BCa patients (Figure 6a), confirming again that EN2 is a PCa‐specific biomarker. Here we applied the 3p3 immunoassay to detect urinary EN2 for the diagnosis of BPH and BCa using the urine samples collected from 15 BPH and 15 BCa patients (Table 1). Figure 6b and Tables S4a and S4b show that EN2 was never detected in the urine of BPH and BCa patients and only detected in the PCa patient urine. When the 3p3 immunoassay to detect urinary PSA was applied for the same BPH and BCa patients, a noticeable amount of PSA was detected in the both BPH and BCa patient urine (Figures 6b and S6, Table S4c,d), corresponding to the previous literatures reporting that PSA is present even in the blood of BPH and BCa patients^62^, ^63^ (Table S5a,b). Consequently, the 3p3 immunoassay can selectively discriminate PCa from other related diseases such as BPH and BCa, indicating precise *in vitro* diagnosis of prostate cancer.

## 3 DISCUSSION

Although urine is an excellent specimen for *in vitro* cancer diagnosis, the urine complexity and variability interfere with the interaction between disease‐specific urinary markers and their cognate antibody probes and hence significantly hampers diagnostic performance. This unique urine environment renders conventional immunoassays (e.g., ELISA, ECLIA, *etc*.) unsuitable any more for *in vitro* cancer diagnosis. Here, we developed an innovative assay methodology, that is, 3p3 immunoassay that is based on cancer marker capture by 3D‐IgG probes in a 3D solution where both 3D‐IgG probes and cancer markers move through omnidirectional diffusion, which remarkably enhances diagnostic sensitivity. The 3D‐IgG probes were constructed by attaching multiple IgG molecules on a single 3D scaffold—genetically modified HBV capsid particle. That is, the surface of HBV capsid particle—consisting of 240 subunit proteins—was modified through genetically inserting tandem SPA_B_ into each of the 240 spike regions, resulting in the localization of 240 copies of (SPA_B_)_2_ on the outermost surface of a single capsid particle. Since SPA_B_ has a specific and strong affinity for Fc region of IgG, all anticancer marker IgG molecules are attached to the SPA_B_‐presenting capsid particle with such an orientation that each Fc region is always perpendicular to the capsid particle surface, resulting in the construction of 3D‐IgG probe. Thus, all antigen‐binding domains of the 3D‐IgG probe become fully available in capturing cancer markers without steric hindrance. As demonstrated in this study, the 3D‐IgG probes are subject to keep a high marker‐capturing capacity even under urine environment where a high concentration of urea and uric acid significantly attenuates the avidity of IgG probes for urinary marker proteins. Moreover, the 3p3 immunoassay is performed in a single step in an assay solution, requiring neither washing steps nor prelabeled reporter probes. Another important advantage is that depending on a physiological concentration range of urinary cancer markers to be detected, the quantity of 3D‐IgG probes are readily controllable in a solution, whereas it is quite difficult to control the amount of immobilized IgG probes on a 2D surface in conventional immunoassays.

Here we applied the 3p3 immunoassay to the detection of urinary EN2, a PCa‐specific marker. PCa is a major health threat to men, recently surpassing the incidence rate of lung cancer, and thus early diagnosis of PCa is critical to increasing survival rate. Although PSA detection has significantly increased the diagnosis rate of PCa over the past 30 years, PSA level also increases in case of other prostate diseases such as infectious and chronic inflammation, BPH, BCa, and other tissue‐associated diseases near prostate. Reportedly, only a limited fraction (~20%) of PSA‐positive subjects are finally diagnosed with PCa, indicating that many individuals without prostate malignancies undergo unnecessary biopsies or treatment. EN2 is only expressed in PCa cells and secreted into the urine—which has been also confirmed in this study—and therefore is a PCa‐specific urinary marker. The 3p3 immunoassay for detecting urinary EN2 is performed simply by adding patient urine, LAA (reducing agent), and BSA to a premade assay solution containing anti‐EN2 3D‐IgG probes adsorbed by Au^3+^, which is quickly followed by three simultaneous events: (1) EN2‐dependent clustering of the 3D‐IgG probes, (2) reduction of adsorbed Au^3+^, and (3) formation of large gold particles emitting dark blue color that is quantified as absorbance. Because only true EN2 can make such a quick formation of large clusters of 3D‐IgG probes and generate optical signals, there is no need of removing false molecules through additional washing steps. The LOD in the urinary EN2 detection by 3p3 immunoassay was about 10 pg EN2/ml urine, low enough to detect urinary EN2 of PCa patients.

In this study, it has been evidently proven that the 3p3 immunoassay shows superior sensitivity and specificity in the urinary EN2 detection—no false‐negative signals for PCa patients (i.e., 100% sensitivity) and no false‐positive signals for other prostate‐related diseases (BPH and BCa) and healthy controls (i.e., 100% specificity), indicating a precise *in vitro* diagnosis of prostate cancer. On the contrary, the 2D immunoassay using commercially available ELISA kit shows far lower sensitivity and specificity. The 3p3 immunoassay also detected PSA in urine, but PSA was also found in the urine of some patients who suffer from other related disorders such as BPH and BCa—exactly corresponding to the previous findings—confirming that PSA is not a reliable marker in PCa diagnosis. Although EN2 is detected as a cancer marker for proof‐of‐concept in this study, the 3p3 immunoassay can be applied to the detection of a variety of other urinary cancer markers, because any antibodies (IgG) can be readily attached to the surface of SPA_B_‐presenting HBVC via specific interaction between SPA_B_ and Fc region of anti‐marker IgG, leading to the construction of 3D‐IgG probes for detecting any cancer markers. Consequently, this simple, innovative approach has successfully tackled the unresolved issues in the sensitive detection of a urinary PCa‐specific marker and thus holds a great potential in opening up a novel urine test‐based clinical route for precise in vitro cancer diagnosis and also pushing urine immunoassay closer to more widespread adoption.

## 4 EXPERIMENTAL METHODS

### 4.1 Cell culture and immunofluorescence microscopy

Human PCa cells (DU‐145 [Cat. No. 30081] and PC‐3 [Cat. No. 21435], KCLB) were cultured in Roswell Park Memorial Institute‐1640 Medium supplemented with 10% (v/v) fetal bovine serum (FBS), 100 U/ml penicillin, and 100 U/ml streptomycin. Human normal prostate cells (RWPE‐1, ATCC) were cultured in Keratinocyte Serum‐Free Medium supplemented with bovine pituitary extract, human recombinant epidermal growth factor, 10% (v/v) FBS, 100 U/ml penicillin, and 100 U/ml streptomycin. All cell cultures were placed at 37°C under a humidified 5% CO_2_ atmosphere. Mycoplasma contamination was tested, and all cell lines were found to be free of mycoplasma.

To measure EN2 and PSA expression in cancer and normal cells, DU‐145 (1 × 10^5^ cells/dish), PC‐3 (1 × 10^5^ cells/dish), and RWPE‐1 (1 × 10^5^ cells/dish) cells were separately seeded in a 35‐mm confocal dish and cultured for 24 h. Each cell culture was rinsed with PBS (pH 7.4), fixed with 4% (v/v) paraformaldehyde (PFA), and incubated at room temperature for 15 min. The cells were then rinsed with PBS three times and stained with 4′,6′‐diamidino‐2‐phenylindole hydrochloride (DAPI). Then, rabbit anti‐human EN2 polyclonal antibody (IgG) conjugated to FITC (Cat. No. ORB466524; Biorbyt LLC, Cambridge, UK) or anti‐human PSA polyclonal antibody (IgG) conjugated to PE (Cat. No. MBS6494820, MyBiosource) was added to the each of DU‐145, PC‐3, and RWPE‐1 cells for 15 min. The cells were rinsed with PBS and imaged using a confocal laser scanning microscope (LSM 700; Carl Zeiss, AG, Jena, Germany). Fluorescence was measured at the following excitation (Ex) and emission (Em) wavelengths: DAPI (Ex/Em: 405/488 nm), FITC (Ex/Em: 495/519 nm), and PE (Ex/Em: 566/574 nm).

### 4.2 Immunoblotting analysis

To determine whether EN2 or PSA is expressed in the aforementioned cell lines, 12% sodium dodecyl sulfate‐polyacrylamide gel electrophoresis (SDS‐PAGE) and Western blotting were performed using the cell lysates. The proteins separated in the SDS‐PAGE gel were transferred to a polyvinylidene fluoride membrane and subjected to Western blotting. The primary antibodies were rabbit anti‐human EN2 polyclonal antibody (IgG) (Cat. No. TA32493; Origene) or rabbit anti‐human PSA polyclonal antibody (IgG) (Cat. No. 70‐XG69; Fitzgerald), and mouse anti‐β‐actin monoclonal antibody (IgG) (Cat. No. sc‐517582; Santa Cruz Biotechnology, Dallas, TX, USA). The secondary antibodies were HRP‐conjugated goat anti‐rabbit antibody (Cat No. 31460; Pierce, Rockford, IL, USA) and HRP‐conjugated goat anti‐mouse antibody (Cat No. 31430; Pierce). The detailed procedures for Western blot analysis have been described in our previous studies.^64^

To detect EN2 in the urine of PCa, BPH, and BCa patients as well as healthy controls, the urine samples were first centrifuged at 10,000 rpm for 5 min to remove intact cells and cellular debris. Then 12% SDS‐PAGE was performed, followed by transferring the separated proteins in the gel to a polyvinylidene fluoride membrane and subsequently performing Western blotting using rabbit anti‐human EN2 polyclonal primary antibody (IgG) (Cat. No. TA3249; Origene) and HRP‐conjugated goat anti‐rabbit secondary antibody (Cat No. 31460; Pierce).

### 4.3 TEM, EDX, and spectrophotometric analyses

TEM and EDX spectroscopy analyses were performed using a Tecnai 20 operated at 200 kV (FEI, Hillsboro, OR, USA). TEM samples were prepared by placing one drop colloidal solution onto 200 square mesh copper grids with carbon film (Electron Microscopy Science, Hatfield, PA, USA) followed by negative staining with 2% (w/v) uranyl acetate solution and air‐drying for 1 h. TEM images were obtained using a charge coupled device (CCD) camera and FEI‐imaging software installed in the Tecnai 20. For EDX spectrum, the detected signal was plotted as a function of characteristic energy. Absorbance spectra of 3p3 immunoassay solutions was analyzed with a TECAN Microplate Reader (Infinite M200 Pro; TECAN, Zürich, Switzerland).

### 4.4 Estimation of dissociation constants ( *K* _d_) in the binding between anti‐EN2 IgG probes and EN2 in urine and serum

The dissociation constants (*K*_d_) of anti‐EN2 3D‐IgG probe and anti‐EN2 IgG antibody in the binding to standard EN2 protein in serum and urine were estimated as follows: each well of a Nunc Maxisorp ELISA plate (Cat. No. 44‐2404‐21; Thermo Fisher Scientific) was coated with 2 μg/ml anti‐EN2 3D‐IgG probes (or 2 μg/ml anti‐EN2 antibody) and incubated at 4°C overnight. The plate was rinsed with PBS and 0.05% (v/v) Tween 20, and each well was blocked with 200 μl superblocking buffer (Cat. No. 37515; Thermo Fisher Scientific) for 4 h. Then standard Myc‐tagged EN2 in urine was added to each well, and the plate was sealed and incubated at 4°C overnight. The binding between the anti‐EN2 3D‐IgG probes (or anti‐EN2 IgG antibodies) and the standard EN2 protein was detected by adding mouse HRP‐conjugated anti‐Myc IgG (Cat. No. R951‐25; Invitrogen, Waltham, MA, USA) and 100 μl of 1X TMB (3,3′,5,5′‐tetramethylbenzidine) solution to each well and by stopping the reactions with 1 M H_2_SO_4_ after 1 h. Absorbance was measured at 450 nm in a microplate reader (Infinite M200 PRO; TECAN). The experimental procedure above was repeated with the EN2 standard in serum.

### 4.5 2D immunoassays using commercial ELISA kits (ELISA‐EN2 and ELISA‐PSA)

Commercial EN2 ELISA kit (Human) (Cat. No. OKCD09228; Aviva Systems Biology) and PSA ELISA kit (Human) (Cat. No. MBS355412; MyBiosource) were used to detect urinary EN2 and PSA of 30 PCa, 15 BPH, and 15 BCa patients as well as 30 healthy controls.

### 4.6 Activity assay of enzyme and fluorescent proteins in urine and serum

Each well of a Nunc Maxisorp ELISA plate (Cat. No. 44‐2404‐21, Thermo Fisher Scientific) was coated with 100 ng/ml HRP‐conjugated avidin (Cat. No. OOMA00043; Aviva Systems Biology) at 4°C overnight. The plate was then rinsed with PBS buffer and 0.05% (v/v) Tween 20. Ten healthy serum and 10 healthy urine samples were added to each well, and the plate was sealed and incubated at room temperature for different time periods. Then the plate was rinsed again with PBS and 0.05% (v/v) Tween 20. Then 1X TMB solution was added to each well and the reaction was stopped with 1 M H_2_SO_4_. The assay signals (absorbance at 450 nm) were measured at each time point in a microplate reader (Infinite M200 PRO; TECAN).

To measure fluorescence intensity, 100 μl of fluorescent protein (EGFP, mOrange, or DsRed, 20 μg/ml) was added to each well of Nunc F96 Micro Well Black Polystyrene plate (Cat. No. 137101, Thermo Fisher Scientific). Then 100 μl of either healthy serum or healthy urine was added to each well, and the plate was sealed and incubated at room temperature for different time periods. The fluorescence intensities were measured at the following excitation (Ex) and emission (Em) wavelengths: EGFP (Ex/Em: 475/509 nm), mOrange (Ex/Em: 520/562 nm), and DsRed (Ex/Em: 541/596 nm) in a microplate reader (Infinite M200 PRO; TECAN, Zürich, Switzerland).

### 4.7 3p3 immunoassay to detect urinary EN2 and PSA

#### 4.7.1 Preparation of 3D‐IgG probes

For the 3p3 immunoassay of EN2 in patient urine, anti‐EN2 3D‐IgG probes were prepared as shown in Figure S1. Assembly PCR with the appropriate primers was used to prepare two clones coding for *NH*_*2*_‐*Nde*I‐hexahistidine(H_6_)‐HBVcAg(1–78)‐G_4_SG_4_T‐*Xho*I‐*COOH* and *NH*_*2*_‐*Bam*HI‐G_4_SG_4_S‐HBVcAg(81–149)‐*Hin*dIII‐*COOH*, where HBVcAg represents the subunit protein (core protein) of HBV capsid. The clones, *NH*_*2*_‐*Xho*I‐SPA_B_‐*Eco*RI‐*COOH* and *NH*_*2*_‐*Eco*RI‐SPA_B_‐*Bam*HI‐*COOH* were also prepared to replace the P79A80 of HBVcAg with the tandem repeat of the B domain (209–271) of *Staphylococcal* Protein A (SPA_B_). Sequential clone ligation was performed to construct the expression vector pT7‐HBVC‐SPA encoding *NH*_*2*_‐H_6_‐HBVcAg(1–78)‐G_4_SG_4_T‐(SPA_B_)_2_‐G_4_SG_4_‐HBVcAg(81–149)‐*COOH* (hereafter, H_6_‐SPA_B_‐capsid). DNA sequencing was conducted with the gel‐purified pT7‐HBVC‐SPA, *Escherichia coli* strain BL21(DE3) [F^−^ *omp*T*hsd*S_B_(rB^−^mB^−^)] was transformed with pT7‐HBVC‐SPA, and ampicillin‐resistant transformants were selected. The experimental procedures used for gene expression and purification of the H_6_‐SPA_B_‐capsid were described in our previous report.^64^ Rabbit anti‐EN2 polyclonal IgG antibodies (Cat. No. TA324936; Origene) (200 μl, 10 mg/L in PBS buffer [2.7 mM KCl, 137 mM NaCl, 2 mM KH_2_PO_4_, and 10 mM Na_2_HPO_4_ at pH 7.4]) were added to the H_6_‐SPA_B_‐capsid (800 μl, 12.5 mg/L in Tris buffer [50 mM Tris–HCl and 500 mM NaCl at pH 7.0]), followed by incubation with mild stirring at 4°C for 12–16 h to make the anti‐EN2 3D‐IgG probes. For the preparation of anti‐PSA 3D‐IgG probes, goat anti‐PSA polyclonal IgG antibodies (Cat. No. 70‐XG69; Fitzgerald) were used instead of rabbit anti‐EN2 polyclonal IgG antibodies above.

#### 4.7.2 Preparation of preassay solution and 3p3 immunoassay

To prepare the preassay solution, Au^3+^ ion solution containing 0.4% (w/v) HAuCl_4_ (Cat. No. 254169; Sigma Aldrich Corp., St. Louis, MO, USA) in 500 μl PBS (pH 7.4) was mixed with anti‐EN2 or anti‐PSA 3D‐IgG probes, leading to the adsorption of Au^3+^ ions on the 3D‐IgG probes. Consequently, in the preassay solution, Au^3+^ ions are present in the form of both adsorbed and free ions. To perform the 3p3 immunoassay, 24 μl preassay solution, 4 μl bovine serum albumin (BSA, Cat. No. BAC65, Equitech‐Bio Inc., Kerrville, TX, USA) solution (13% [w/v] in PBS [pH 7.4]), 4 μl urine of patient or healthy donor, and 8 μl LAA (a reducing agent) (Cat. No. A7506; Sigma Aldrich Corp.) (0.7% [w/v] in PBS [pH 7.4]) were added to each well of a 384‐well plate (Cat. No. 3680; Corning Inc., Corning, NY, USA), immediately followed by measuring absorbance at 620 nm (OD_620_ for EN2) or 585 nm (OD_585_ for PSA) every 10 min at room temperature using an Infinite M200 Pro Microplate Reader (TECAN). OD_620_ or OD_585_ is the absorbance by large gold particles formed through the LAA‐mediated reduction of free and adsorbed Au^3+^ ions, the latter being adsorbed on the EN2‐ or PSA‐induced large clusters of 3D‐IgG probes. Since both free and adsorbed Au^3+^ ions are critical to the formation of large gold particles, the free Au^3+^ ions in the preassay solution must not be adsorbed to BSA added to the preassay solution. To prevent the free Au^3+^ ions from adsorbing to BSA, 13% (w/v) BSA (30 ml in PBS [pH 7.4]) was mixed with NiCl_2_ (0.039 g, Cat. No. 451193, Sigma Aldrich Corp.) with mild stirring at 4°C, prior to being added to the preassay solution.

#### 4.7.3 Preparation of urine samples

Urine samples from PCa patients were provided by Seoul National University Hospital Biomedical Research Institute (Seoul, Republic of Korea). Urine samples from BPH‐ and BCa patients and from healthy controls were donated by Biobank of Chungbuk National University Hospital (Cheongju, Republic of Korea) and Biobank of Chungnam National University Hospital (Daejeon, Republic of Korea), respectively. Standard EN2 or PSA urine solutions containing recombinant human EN2 (rEN2) or recombinant human PSA (rPSA) were also prepared as follows: 1 μl rEN2 or rPSA (0.05 mg/ml; Cat. No. TP‐311220; Origene) was added to 999 μl healthy donor urine, and the resulting solution was serially diluted with healthy urine to prepare standard rEN2 or rPSA urine solutions in the concentration range of 0.01–20 ng/ml. All experimental procedures were performed in accordance with the Guidelines for Care and Use of Human Biological Materials of Korea University that were approved by the Institutional Review Board of Korea University.

## AUTHOR CONTRIBUTIONS

**Hye Hyun Kim:** Conceptualization (equal); data curation (equal); investigation (equal); methodology (equal); validation (equal); visualization (equal); writing – original draft (equal); writing – review and editing (supporting). **Ok Jeong Moon:** Data curation (supporting); investigation (supporting); visualization (supporting). **Yong Hwan Seol:** Data curation (supporting); investigation (supporting); visualization (supporting); writing – original draft (supporting). **Jeewon Lee:** Conceptualization (lead); data curation (equal); methodology (equal); supervision (lead); validation (lead); visualization (equal); writing – review and editing (lead).

## CONFLICT OF INTEREST

The authors declare no conflict of interest.

### PEER REVIEW

The peer review history for this article is available at https://publons.com/publon/10.1002/btm2.10489.

## ETHICS STATEMENT

Work with recombinant DNA and microorganisms was performed according to national requirements (Living Modified Organisms protocol‐LML17‐440).

## Supporting information


**Figure S1.** Construction of 3D‐IgG probes and 3p3 immunoassay solution analyzed by TEM and EDX
**Figure S2.** 3p3 immunoassay of standard PSA spiked in healthy urine
**Figure S3.** Correlation between optical assay signals (OD_620_) and EN2 concentration in the 3p3 immunoassay using a different quantity (10, 50, and 100 mg/L) of anti‐EN2 3D‐IgG probes in preassay solution
**Figure S4.** Urinary EN2 detection by 3p3 immunoassays and urinary PSA detection by ELISA
**Figure S5.** Performance of ELISA in the urinary EN2 detection with varying the urine sample volume from 4 to 100 μl
**Figure S6.** Results of the 3p3 immunoassay to detect urinary EN2 and PSA in PCa‐related diseases (BPH and BCa)
**Table S1.** Serum PSA concentration of 30 PCa patients
**Table S2.** Results of 3p3 immunoassay and ELISA to detect urinary EN2 of PCa Patients and healthy individuals
**Table S3.** Results of 3p3 immunoassay and ELISA to detect urinary PSA of PCa patients and healthy individuals
**Table S4.** Results of 3p3 immunoassay to detect urinary EN2 and PSA of BPH and BCa patients
**Table S5.** Serum PSA concentration of (a) 15 BPH and (b) 15 BCa patients

## Data Availability

The data that support the findings of this study are available from the corresponding author upon reasonable request.
