# Supplementary material for: A simple urine test by 3D‐plus‐3D immunoassay guides precise in vitro cancer diagnosis
Source: Bioeng Transl Med. 2023 Jan 18;8(3):e10489. doi: 10.1002/btm2.10489 (PMC10189436; doi:10.1002/btm2.10489)

Supporting Information for *Bioengineering & Translational Medicine*

A simple urine test by 3D-plus-3D immunoassay guides precise *in vitro* cancer diagnosis

*Hye Hyun Kim, Ok Jeong Moon, Yong Hwan Seol, and Jeewon Lee**

[*] Prof. J. Lee (Corresponding author), H. H. Kim, O. J. Moon, Y. H. Seol

Department of Chemical and Biological Engineering, College of Engineering,

Korea University, Anam-Ro 145, Seoul 136-713 (Republic of Korea)

E-mail: leejw@korea.ac.kr

**Supplementary Figures**

**Figure S1**


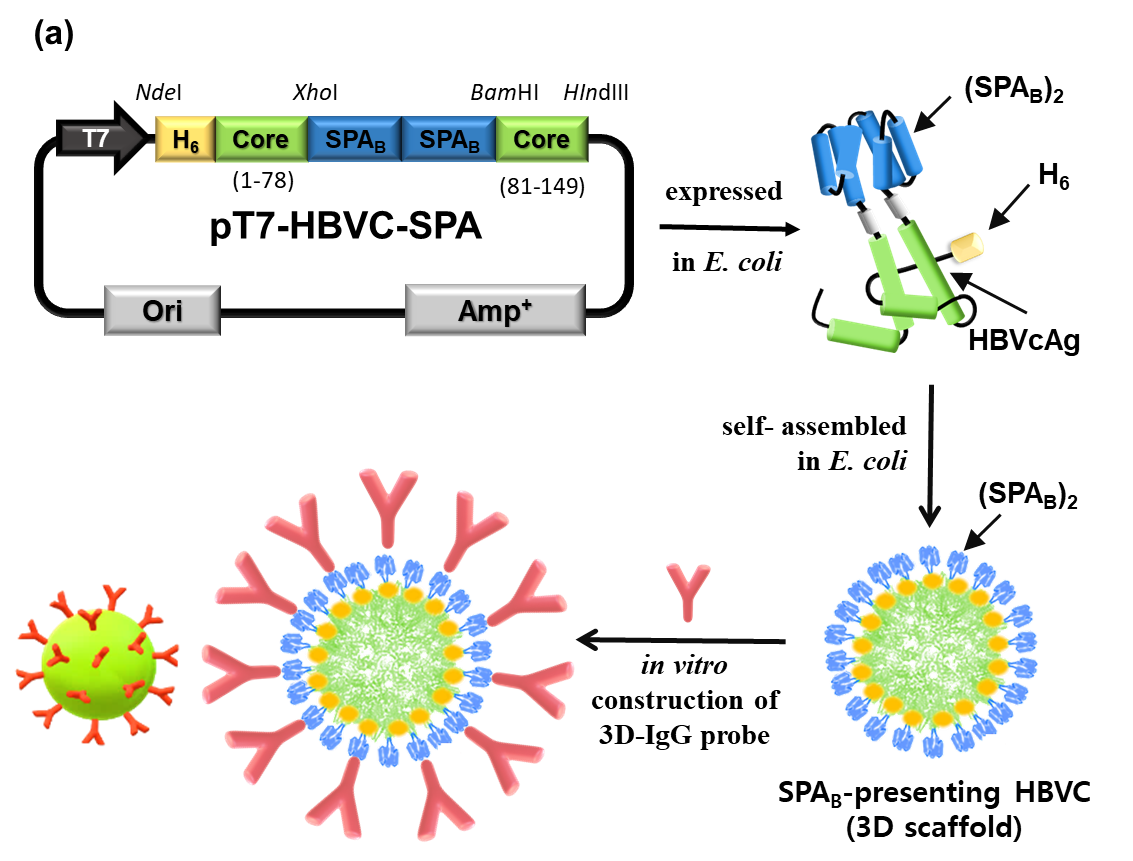


**
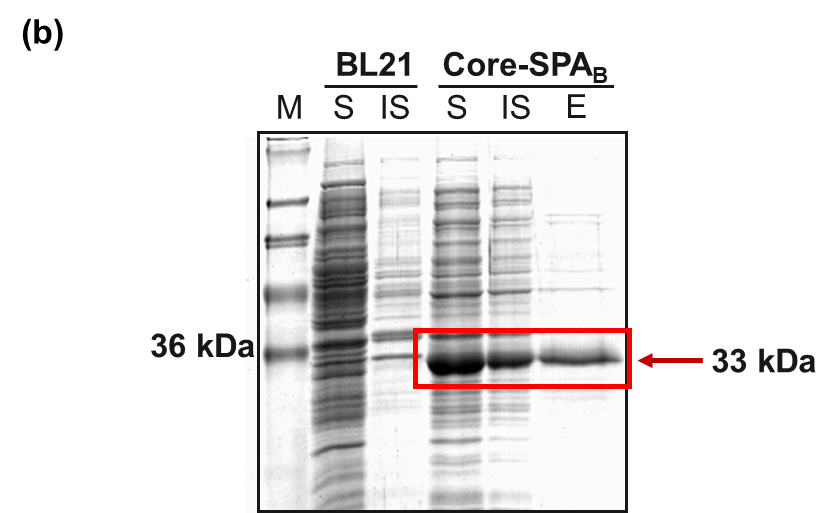
**

**
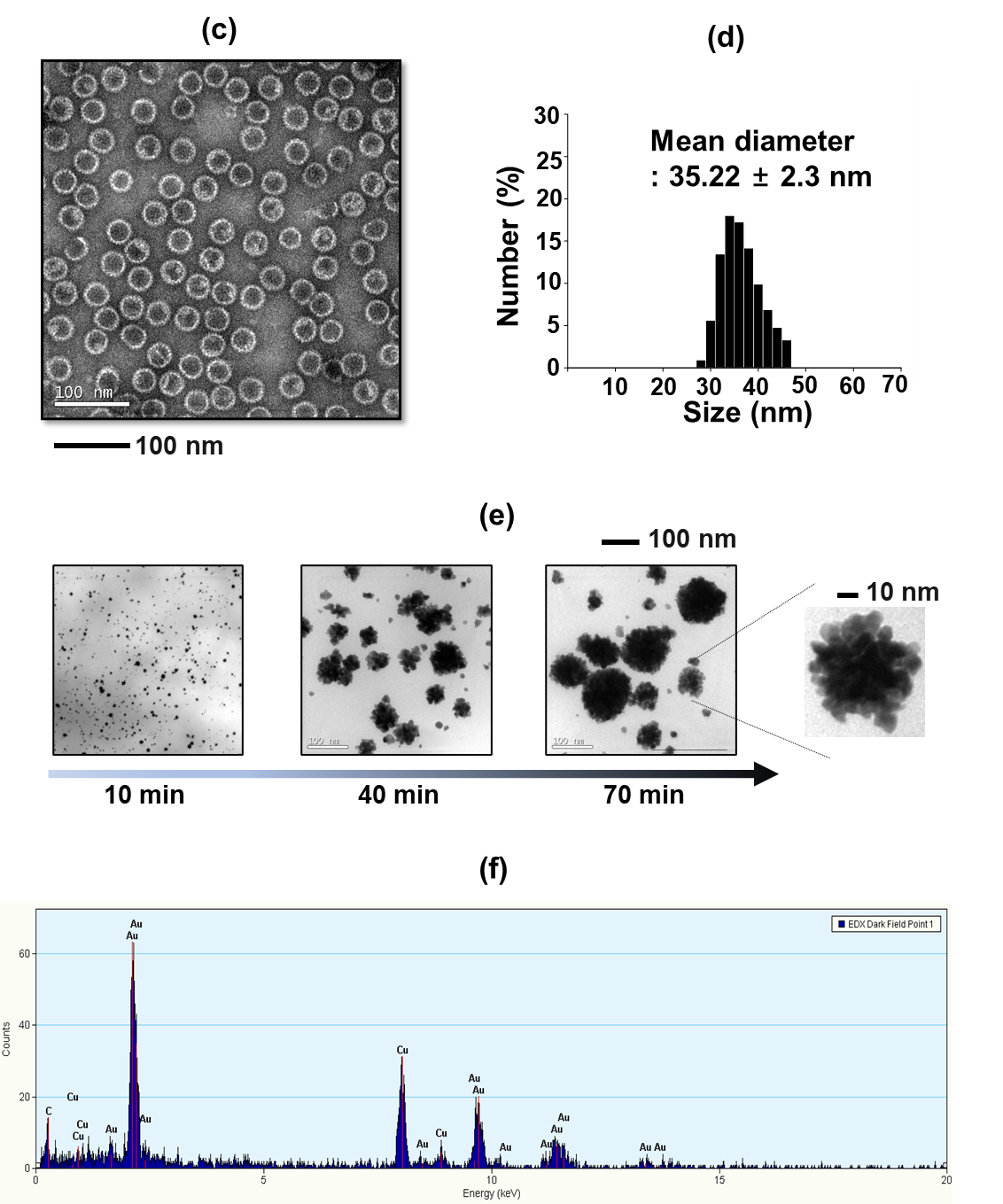
**

**Figure S1 | Construction of 3D-IgG probes. and 3p3 immunoassay solution analyzed by TEM and EDX. (a)** Plasmid expression vector for synthesizing H_6_-SPA_B_-capsid in recombinant *E. coli* and construction of 3D-IgG probe using H_6_-SPA_B_-capsid as a 3D scaffold. **(b)** SDS-PAGE of soluble (S) and insoluble fraction (IS) of recombinant *E. coli* cell lysates and eluted fraction (E) from Ni^+2^-affinity column chromatography of soluble fraction. (M, molecular marker; BL21, wild-type *E. coli* cell lysates). **(c)** TEM image of Ni^+2^ affinity-purified H_6_-SPA_B_-capsid. **(d)** Result of DLS analysis of Ni^+2^ affinity-purified H_6_-SPA_B_-capsid. **(e)** TEM images showing time-course formation of large gold particles in 3p3 immunoassay solution where EN2 in PCa-patient urine is detected using anti-EN2 3D-IgG probes. **(f)** Result of EDX analysis of the 3p3 immunoassay solution of (e).

**Figure S2**


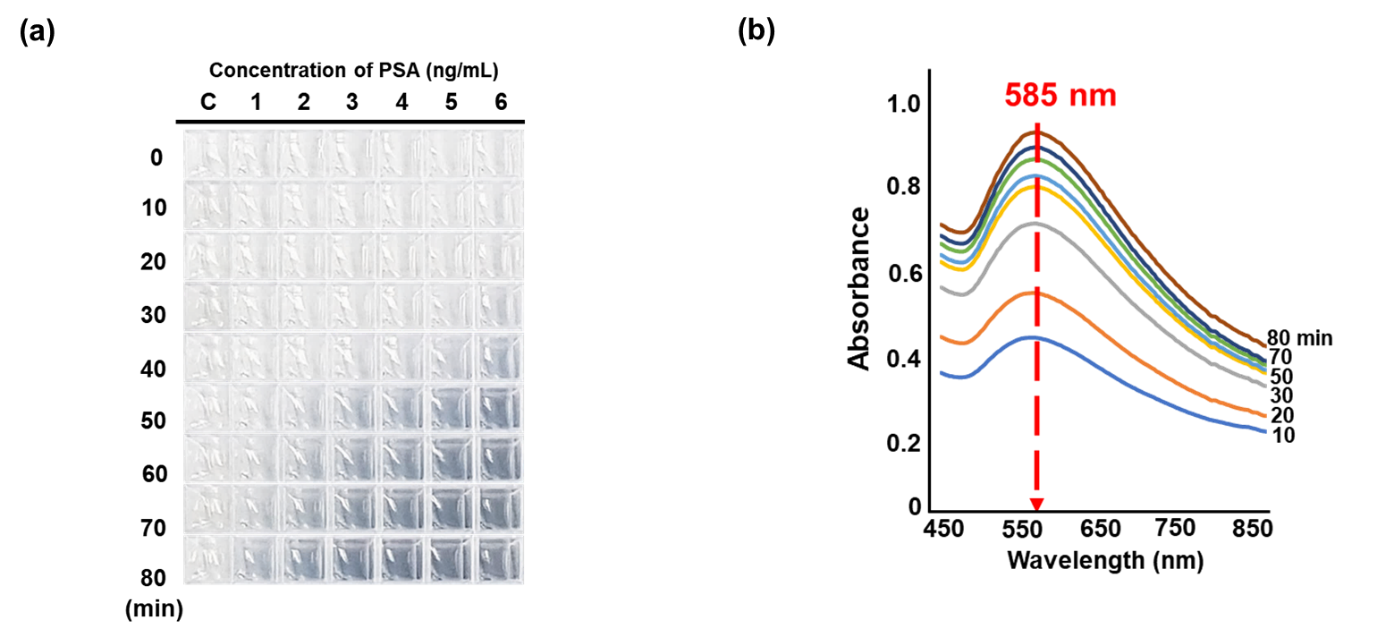


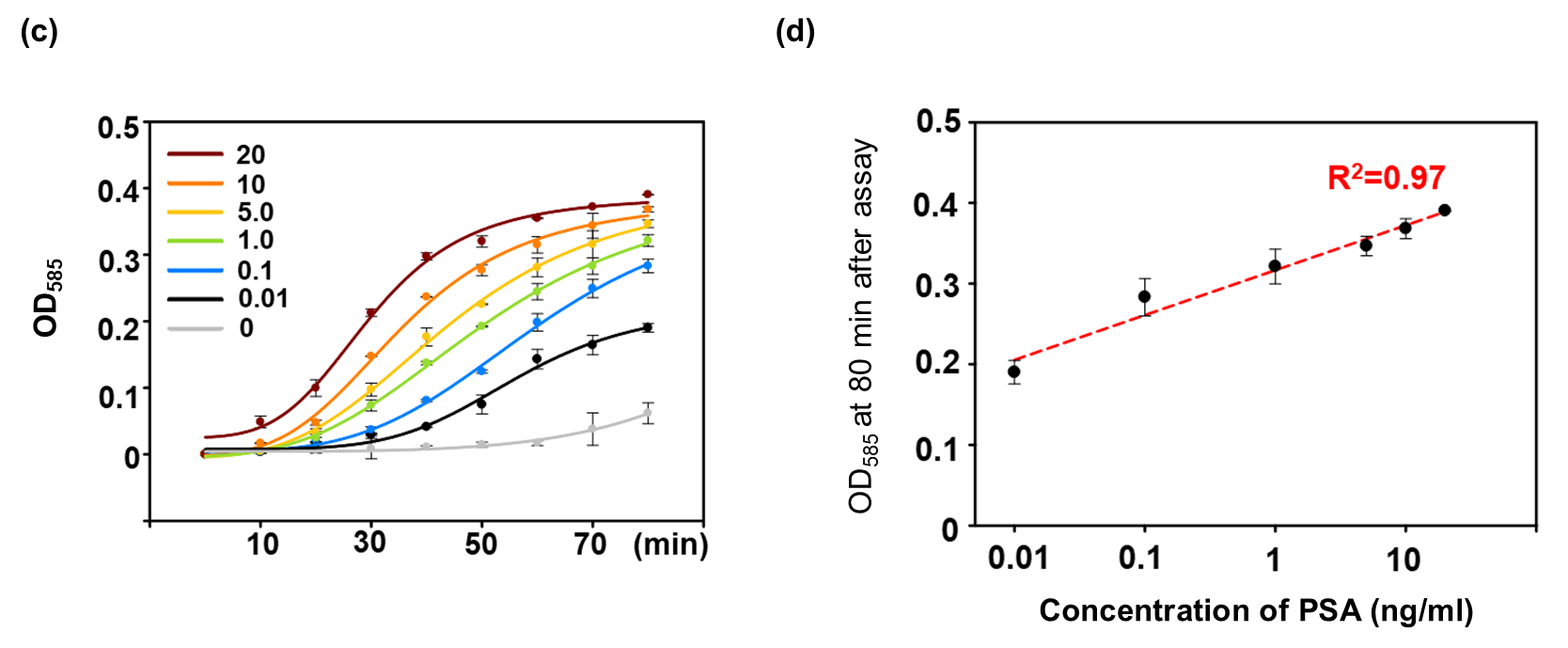


**Figure S2 | 3p3 immunoassay of standard PSA spiked in healthy urine. (a)** Photographic images of 384-well plates, showing time-course change of optical assay signals in the 3p3 immunoassay performed with urine samples of standard PSA at different concentrations (C: 0 ng/mL, 1: 0.01 ng/mL, 2: 0.1 ng/mL, 3: 1.0 ng/mL, 4: 5.0 ng/mL, 5: 10 ng/mL, 6: 20 ng/mL). **(b)** Time-course change of light absorption spectra of sample 6 in (a), showing that maximum light absorption happens always at 585 nm. **(c)** Time-course change of OD_585_ measured for the optical assay signals of (a). **(d)** OD_585_ measured at 80 min after the PSA detection by 3p3 immunoassay of (a) begins.


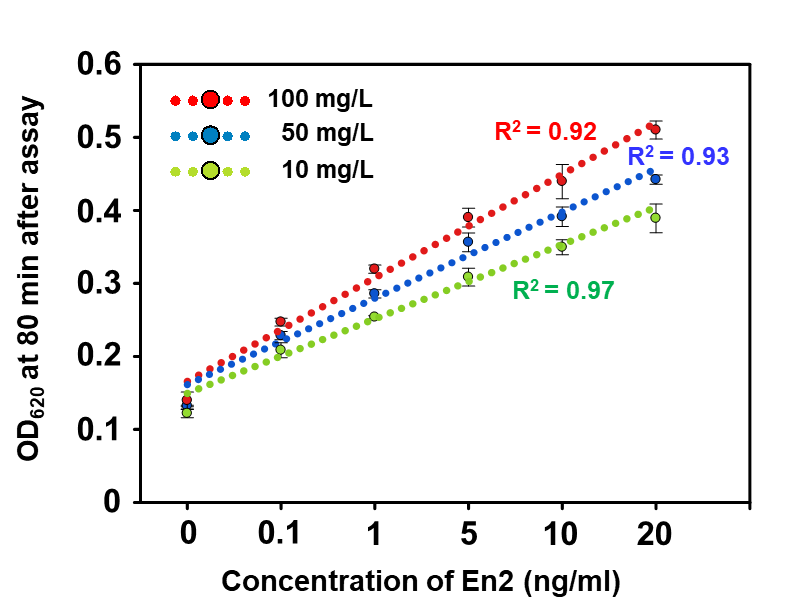
**Figure S3**

**Figure S3 | Correlation between optical assay signals (OD_620_) and EN2 concentration in the 3p3 immunoassay using a different quantity (10, 50, and 100 mg/L) of anti-EN2 3D-IgG probes in pre-assay solution.**

**
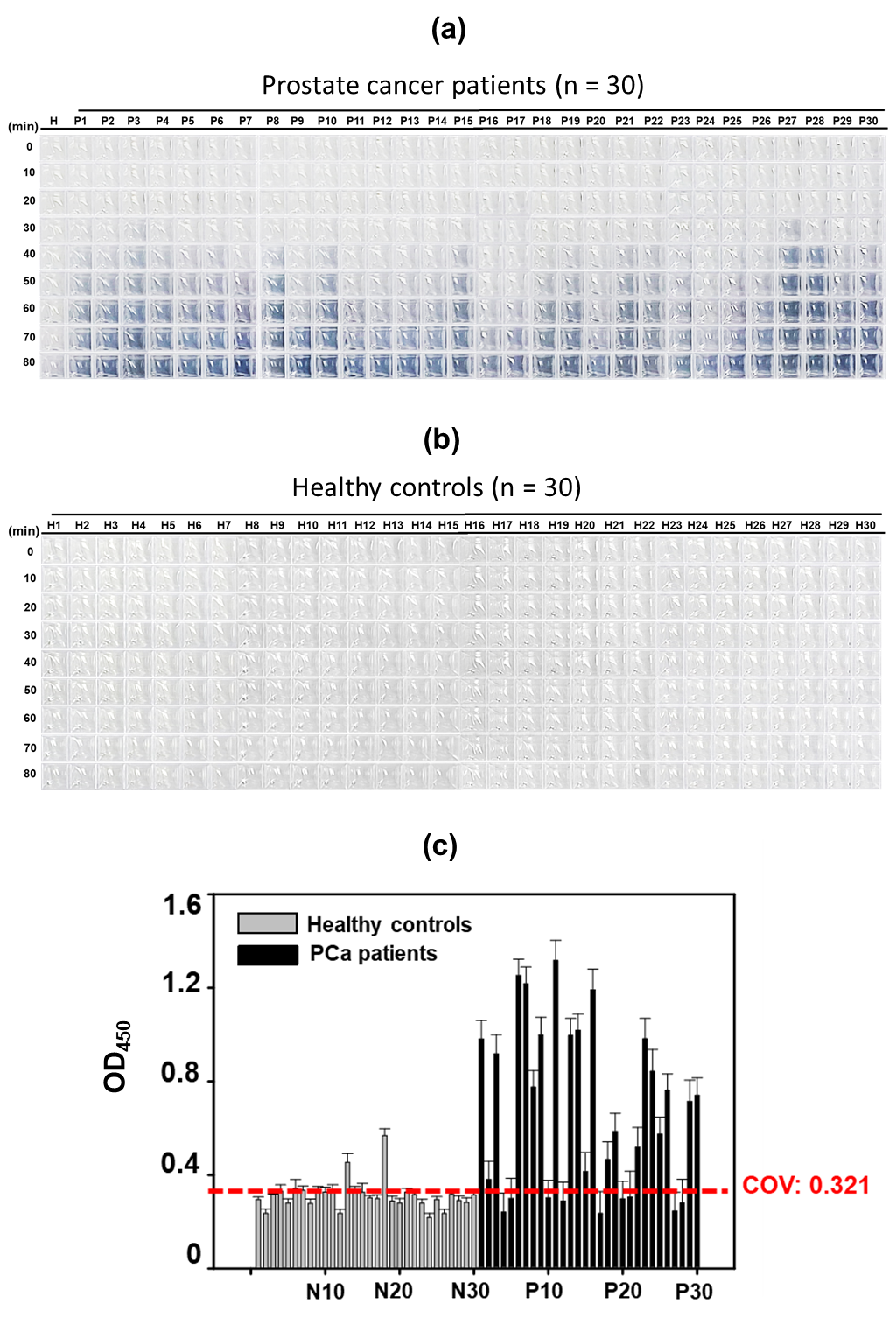
Figure S4**

**Figure S4 | Urinary EN2 detection by 3p3 immunoassays and urinary PSA detection by ELISA. (a)** Photographic images of 384-well plates, showing time-course change of optical assay signals in the 3p3 immunoassay to detect EN2 using 30 PCa-patient urine samples. **(b)** Photographic images of 384-well plates, showing time-course change of optical assay signals in the 3p3 immunoassay to detect EN2 using 30 healthy urine samples. **(c)** Result of ELISA (using a commercial ELISA kit) to detect PSA in the same urine samples of (a) and (b).

**Figure S5**


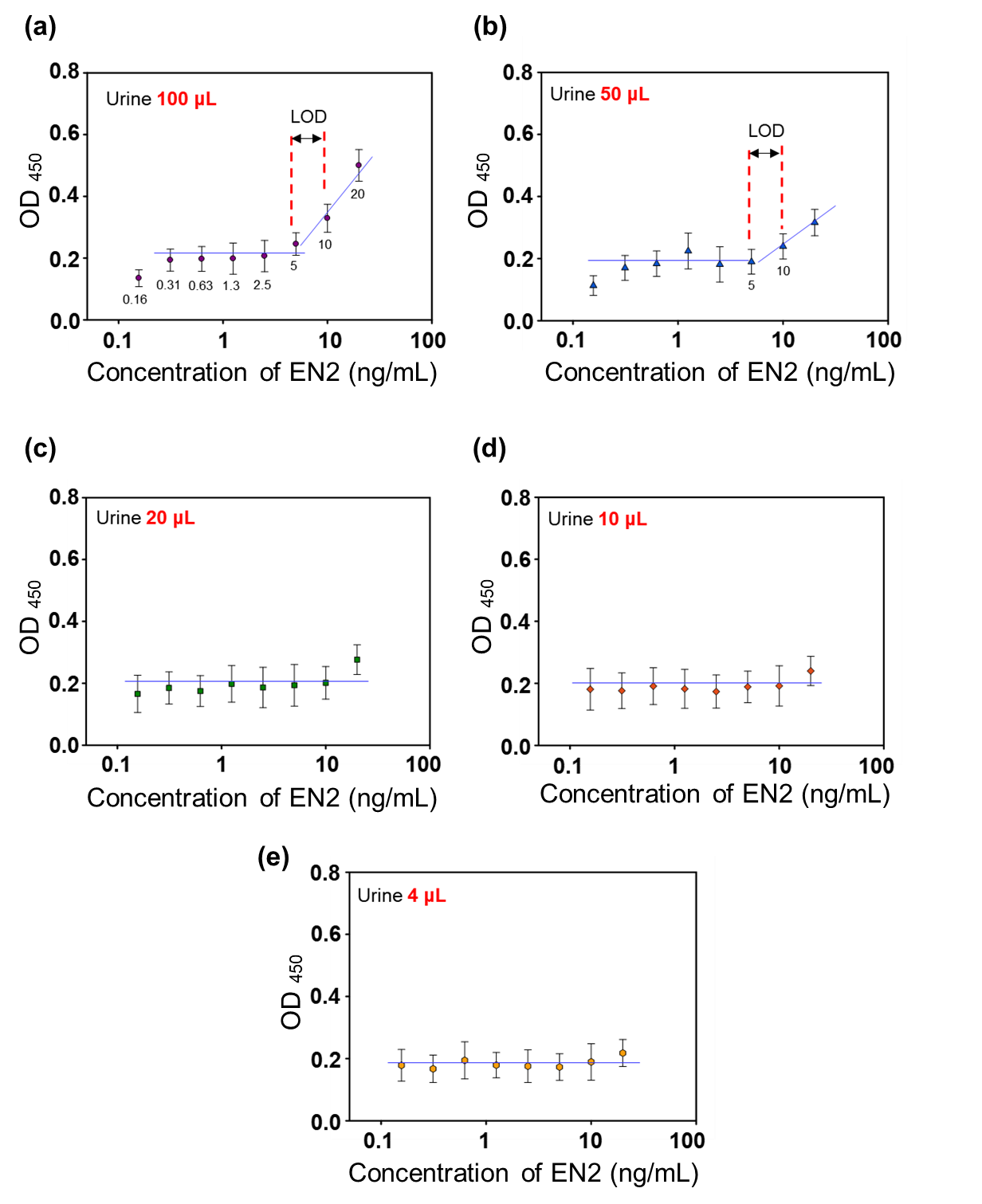


**Figure S5 | Performance of ELISA in the urinary EN2 detection with varying the urine sample volume from 4 to 100 μL.**

**
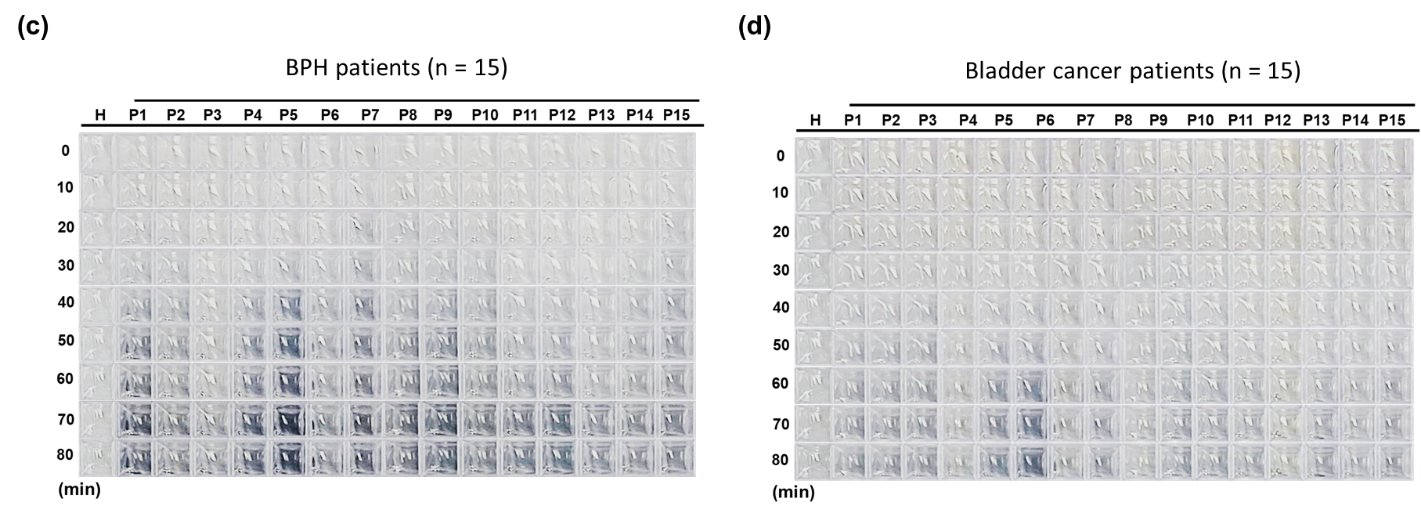

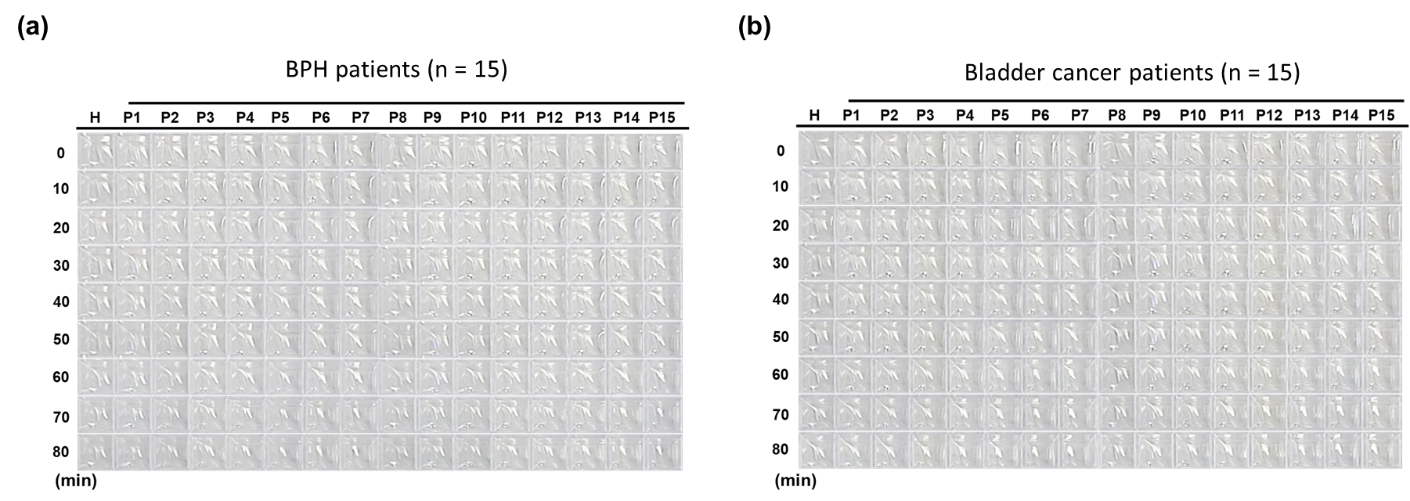
Figure S6**

**Figure S6 | Results of the 3p3 immunoassay to detect urinary EN2 and PSA in PCa-related diseases (BPH and BCa).** Photographic images of 384-well plates, showing time-course change of optical assay signals in the 3p3 immunoassay to detect urinary EN2 using **(a)** 15 BPH- and **(b)** 15 BCa-patient urine samples. Photographic images of 384-well plates, showing time-course change of optical assay signals in the 3p3 immunoassay to detect urinary PSA using **(c)** 15 BPH- and **(d)** 15 BCa-patient urine samples**.**

**Supplementary Tables**

**Table S1. Serum PSA concentration of 30 PCa patients.**

**
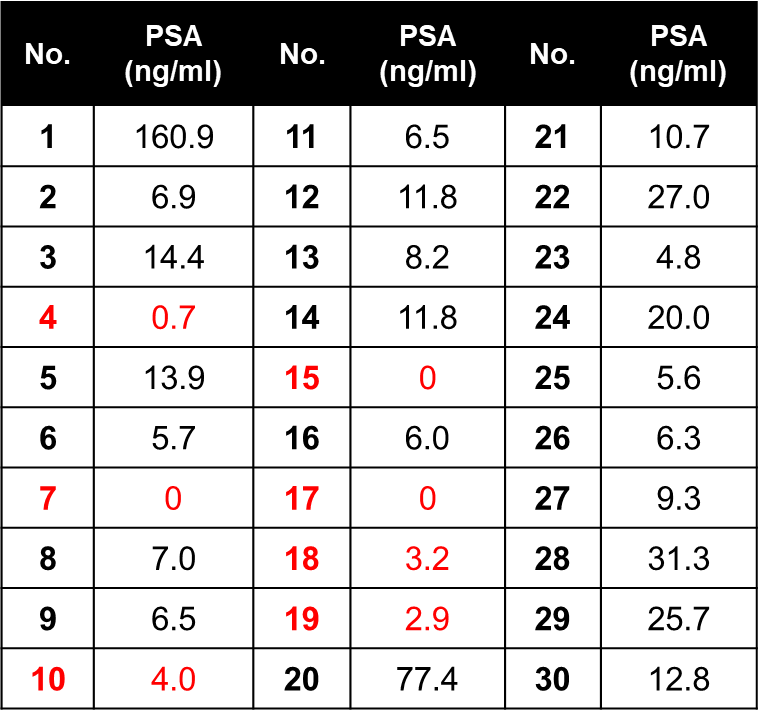
**

**
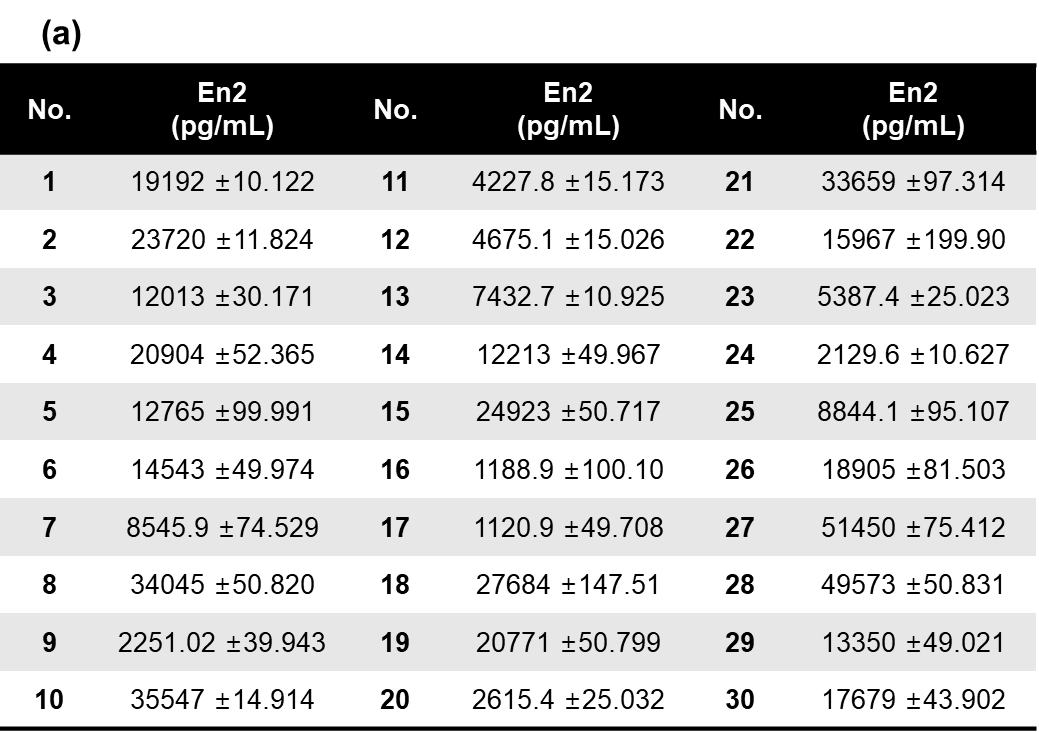

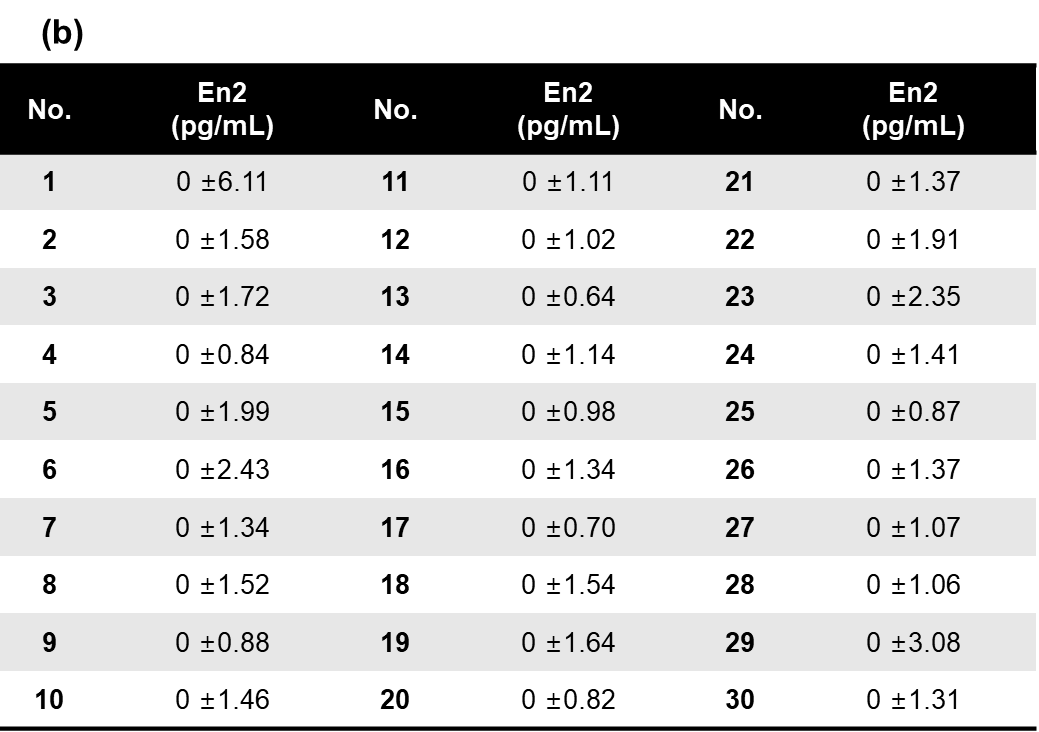
****Table S2. Results of 3p3 immunoassay and ELISA to detect urinary EN2 of PCa Patients and healthy individuals. (a)** 3p3 immunoassay using 30 PCa-patient urine samples. **(b)** 3p3 immunoassay using 30 healthy urine samples. **(c)** ELISA using 30 PCa-patient urine samples. **(d)** ELISA using 30 healthy urine samples.

**
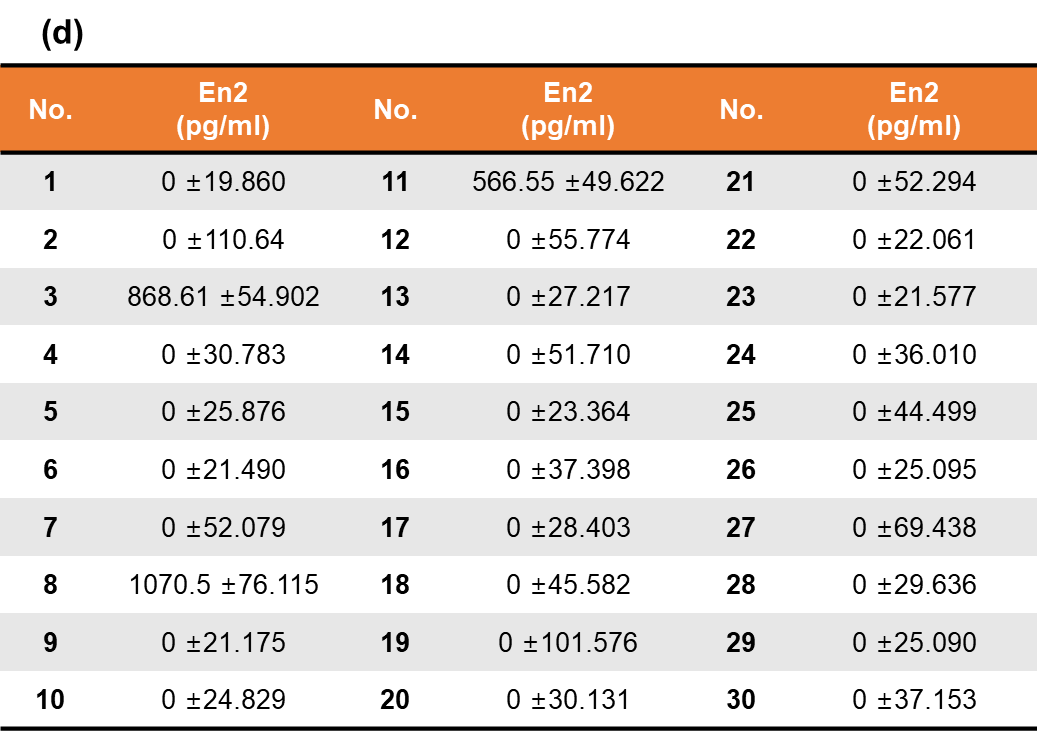

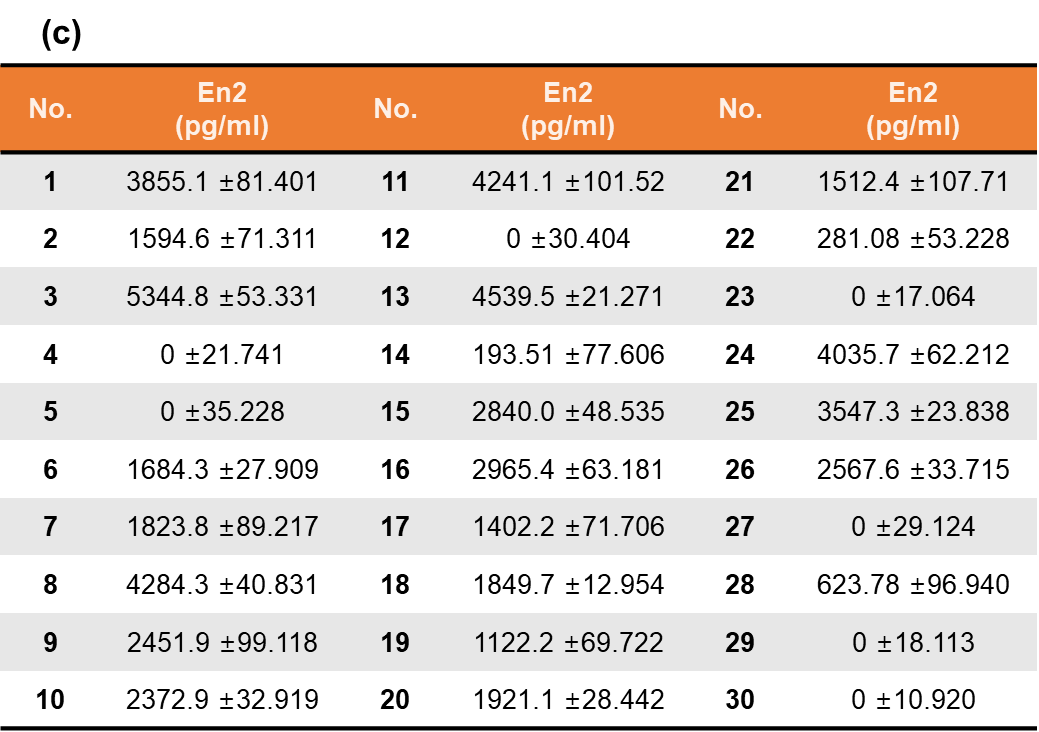
**

**Table S3. Results of 3p3 immunoassay and ELISA to detect urinary PSA of PCa patients and healthy individuals. (a)** 3p3 immunoassay using 30 PCa-patient urine samples. **(b)** 3p3 immunoassay using 30 healthy urine samples. **(c)** ELISA using 30 PCa-patient urine samples. **(d)** ELISA using 30 healthy urine samples.

**
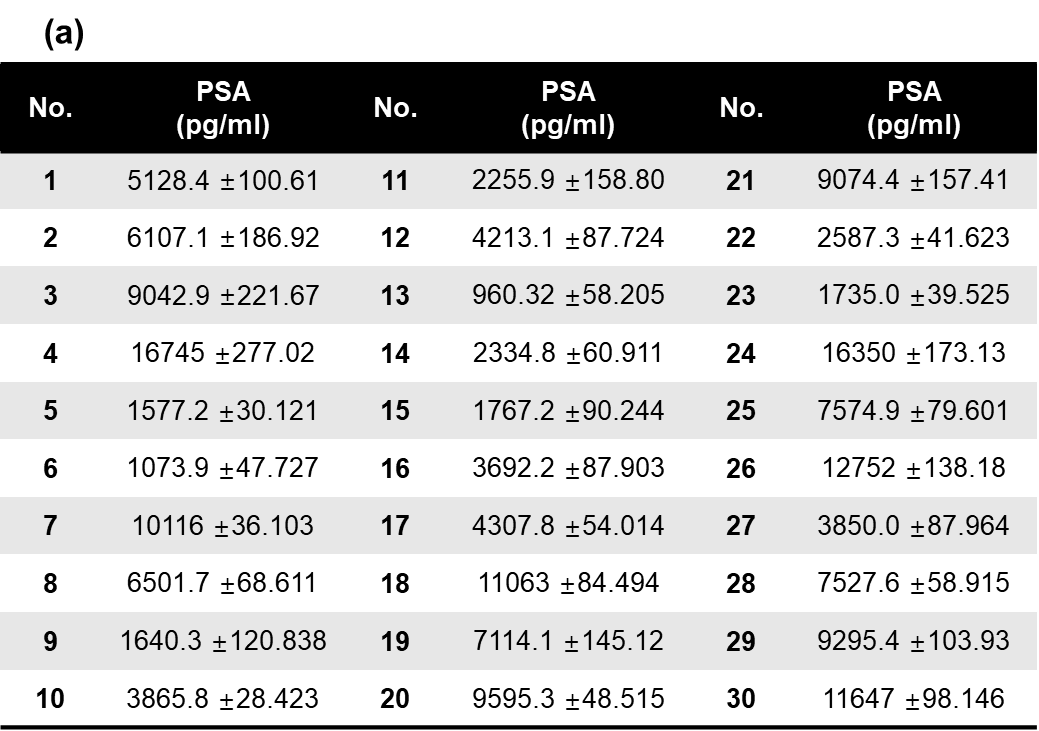
**

**
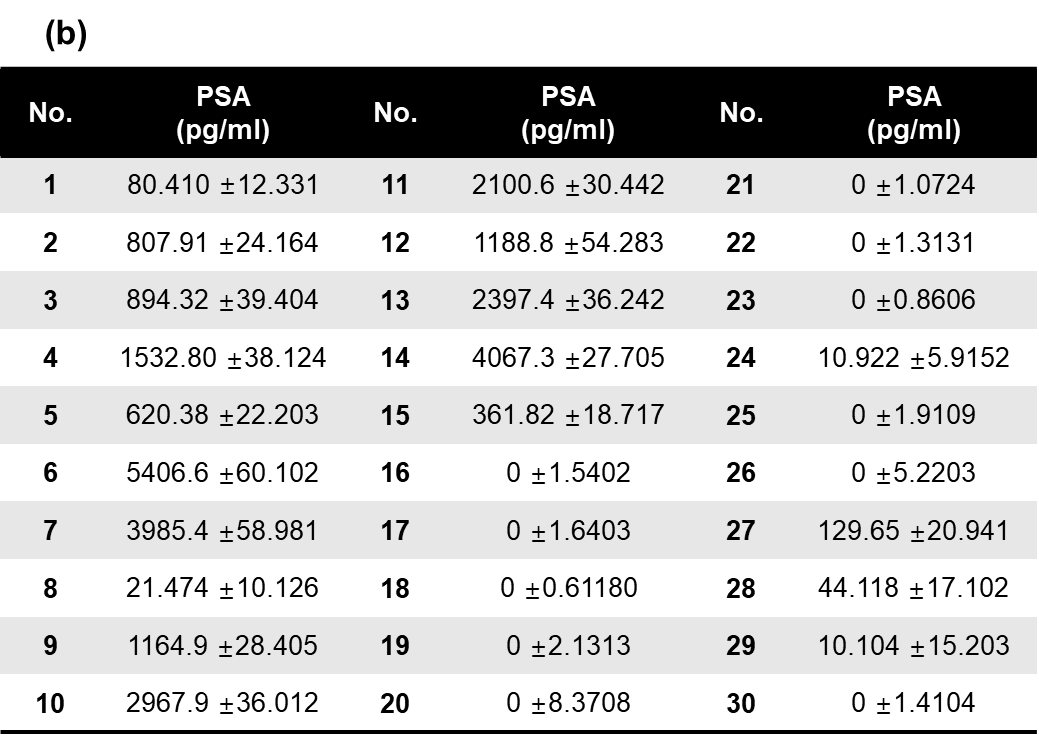
**

**
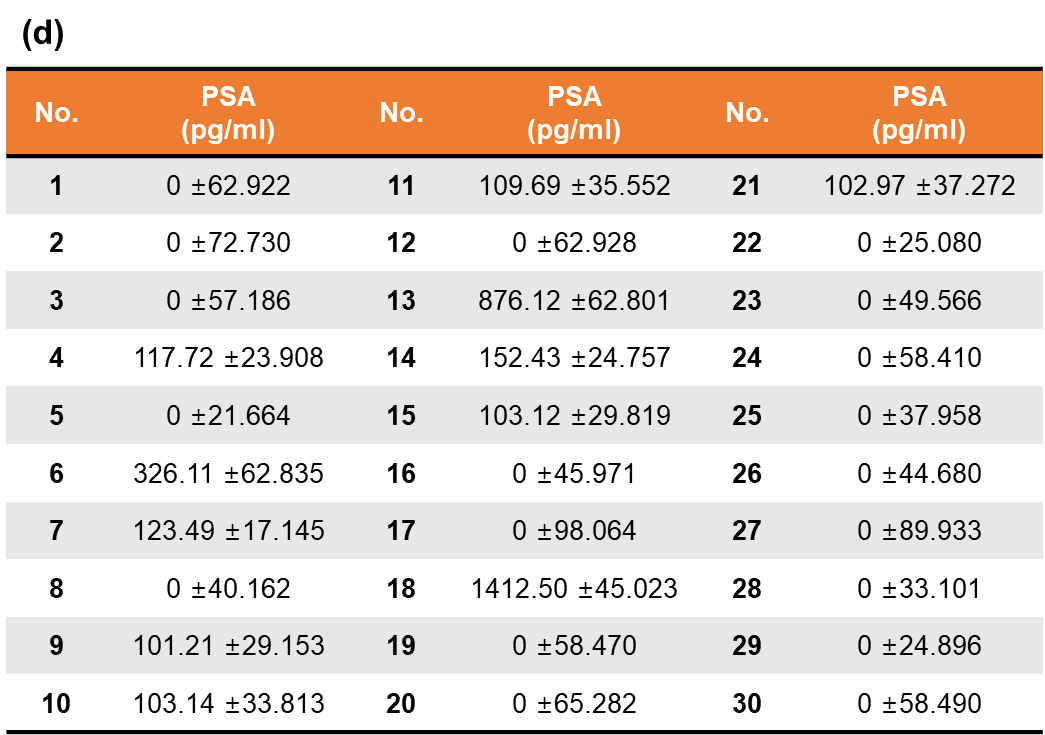
**
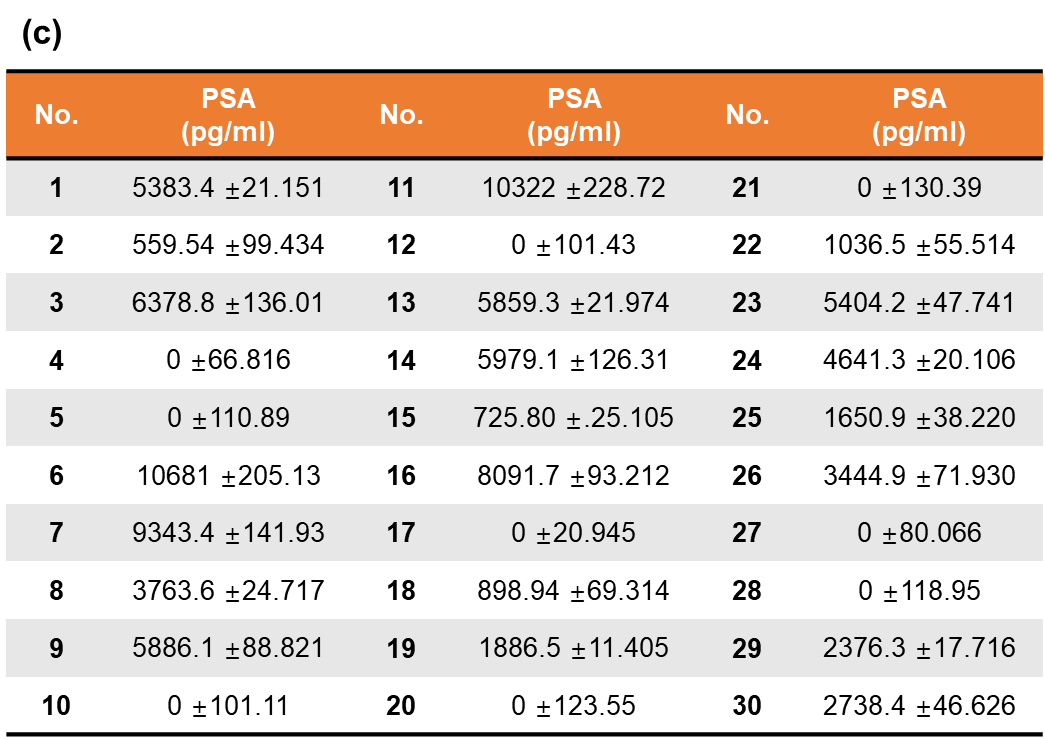


**Table S4. Results of 3p3 immunoassay to detect urinary EN2 and PSA of BPH and BCa patients.** EN2 detection using **(a)** 15 BPH- and **(b)** 15 BCa-patient urine samples. PSA detection using **(c)** 15 BPH- and **(d)** 15 BCa-patient urine samples.

**
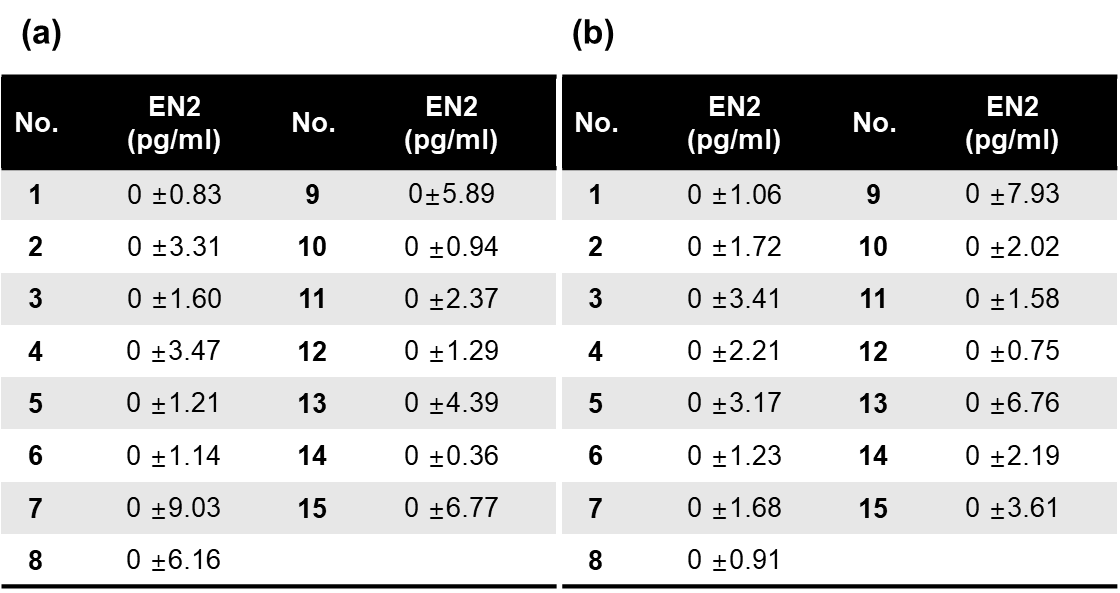

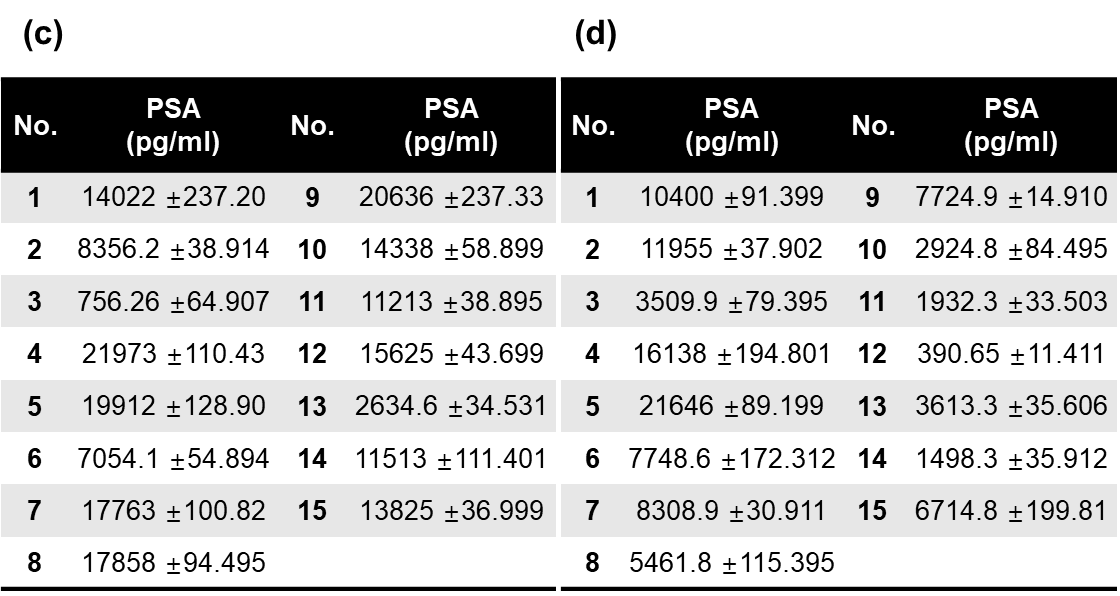
**

**Table S5. Serum PSA concentration of (a) 15 BPH- and (b) 15 BCa patients.**
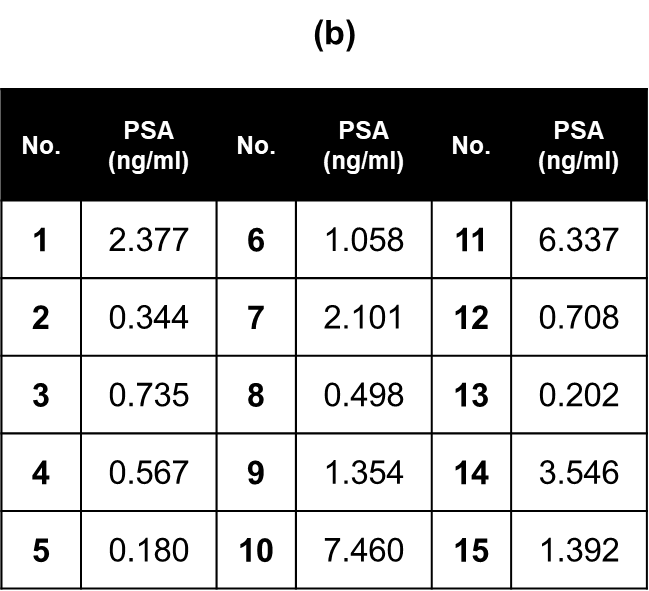

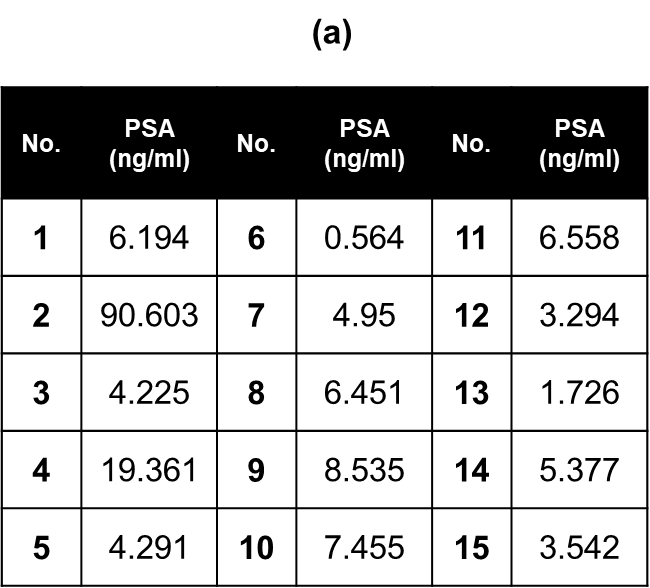

Supplement: Supplementary file 1 — Figure S1. Construction of 3D‐IgG probes and 3p3 immunoassay solution analyzed by TEM and EDX Figure S2. 3p3 immunoassay of standard PSA spiked in healthy urine Figure S3. Correlation between optical assay signals (OD620) and EN2 concentration in the 3p3 immunoassay using a different quantity (10, 50, and 100 mg/L) of anti‐EN2 3D‐IgG probes in preassay solution Figure S4. Urinary EN2 detection by 3p3 immunoassays and urinary PSA detection by ELISA Figure S5. Performance of ELISA in the urinary EN2 detection with varying the urine sample volume from 4 to 100 μl Figure S6. Results of the 3p3 immunoassay to detect urinary EN2 and PSA in PCa‐related diseases (BPH and BCa) Table S1. Serum PSA concentration of 30 PCa patients Table S2. Results of 3p3 immunoassay and ELISA to detect urinary EN2 of PCa Patients and healthy individuals Table S3. Results of 3p3 immunoassay and ELISA to detect urinary PSA of PCa patients and healthy individuals Table S4. Results of 3p3 immunoassay to detect urinary EN2 and PSA of BPH and BCa patients Table S5. Serum PSA concentration of (a) 15 BPH and (b) 15 BCa patients [file BTM2-8-e10489-s001.docx]
